# Waste for Product—Synthesis and Electrocatalytic Properties of Palladium Nanopyramid Layer Enriched with PtNPs

**DOI:** 10.3390/ma17164165

**Published:** 2024-08-22

**Authors:** Magdalena Luty-Błocho, Adrianna Pach, Dawid Kutyła, Anna Kula, Stanisław Małecki, Piotr Jeleń, Volker Hessel

**Affiliations:** 1 AGH University of Krakow, Faculty of Non-Ferrous Metals, Al. A. Mickiewicza 30, 30-059 Krakow, Poland; apach@agh.edu.pl (A.P.); kutyla@agh.edu.pl (D.K.); kula@agh.edu.pl (A.K.); stanmal@agh.edu.pl (S.M.); 2 AGH University of Krakow, Faculty of Materials Science and Ceramics, Al. A. Mickiewicza 30, 30-059 Krakow, Poland; pjelen@agh.edu.pl; 3 School of Chemical Engineering, University of Adelaide, Adelaide 5005, Australia; volker.hessel@adelaide.edu.au

**Keywords:** adsorption, kinetic study, nanopyramides, metals recovery, catalyst, HER

## Abstract

The presented research is the seed of a vision for the development of a waste-for-product strategy. Following this concept, various synthetic solutions containing low concentrations of platinum group metals were used to model their recovery and to produce catalysts. This is also the first report that shows the method for synthesis of a pyramid-like structure deposited on activated carbon composed of Pd and Pt. This unique structure was obtained from a mixture of highly diluted aqueous solutions containing both metals and chloride ions. The presence of functional groups on the carbon surface and experimental conditions allowed for: the adsorption of metal complexes, their reduction to metal atoms and enabled further hierarchical growth of the metal layer on the carbon surface. During experiments, spherical palladium and platinum nanoparticles were obtained. The addition of chloride ions to the solution promoted the hierarchical growth and formation of palladium nanopyramids, which were enriched with platinum nanoparticles. The obtained materials were characterized using UV–Vis, Raman, IR spectroscopy, TGA, SEM/EDS, and XRD techniques. Moreover, Pd@ROY, Pt@ROY, and Pd-Pt@ROY were tested as possible electrocatalysts for hydrogen evolution reactions.

## 1. Introduction

The growing amount of waste solution rich in heavy metals is a global problem, which threatens the devastation of the natural environment and living organisms [1,2,3]. But if we look at it from the other side, it also can be a potential source for the recovery of valuable metals. The recovery of metals from the platinum group metals (PGM) should be considered especially. Recovered PGM, due to their unique chemical properties, can be treated again as input material for, e.g., catalytic purposes, as one of the “Waste for Product” (W4P) conceptions.

W4P conception has a lot of benefits, which are the future of the industry in terms of the possibility of reusing waste, containing a low content of valuable metals. Pach et al. [4] indicate that a low concentration of platinum and palladium ions in the solution was the ideal composition for the synthesis of a bimetallic electrocatalyst for HER, which fits into the W4P approach. Moreover, there is economics behind it, which relates to depleting natural resources and rising metal prices. Thus, in the future, W4P conception for metal recovery might be one of the best solutions promoting sustainable economic development. It is also justified to find solutions that will allow the recovery of metals contained in waste solutions and their processing into a finished product. It is necessary to select the correct recovery method that will synergistically support the process of producing the final product. Taking into account the catalyst production process, catalytic support is needed. Therefore, it is justified to select adsorption processes and appropriate sorbents that, on the one hand, will be suitable for “pulling out” metal ions and, on the other hand, will be a catalyst carrier.

Considering the existing recovery methods described in the literature [5,6,7,8], it seems that the most simple and often used method is the adsorption process [9,10]. For heavy metal adsorption, various sorbents are used, e.g., TiO_2_ [11] or their modified form [12,13,14], ZnO [15,16] or modified zinc oxide [17,18], chitosan [19,20], or chitosan in combination with other compounds [21,22], activated carbon (AC) [23,24], etc. AC is most used, due to the high chemical resistance, the large specific surface area reached in surface groups, and high porosity. Consequently, the adsorption capacity of AC makes it a top material used for metal recovery [25] as well as catalyst carrier [26,27]. However, the diversity of activated carbons makes it necessary to select the appropriate material that will meet the expectations of the W4P approach.

Considering PGM recovery, an adsorption process using activated carbon was successfully applied for palladium and platinum recovery from an acidic, mild acidic solution. For this purpose, different types of AC were used such as NORIT [28,29,30], Organosorb [31], activated carbon fibers (ACF) [32], obtained from bio-waste [33], etc. The efficiency of activated carbon depends on its surface properties and its ability for bond formation between metal ions and surface groups, which are active in this process. Then, adsorbed material can be recovered in the form of ions or (what is in most cases) can be next reduced to metal. This so-called “natural behavior” of activated carbon makes this material a great catalyst carrier. Process catalyst synthesis and deposition on the carrier can be carried out in batch (impregnation process) [34] or in the microreactor system, which enhances the process of metal deposition [35,36]. In this context, the morphology of obtained materials and their properties can be interesting considering the W4P approach. Luty-Błocho et al. [35] obtained Pd nanoparticles of different sizes (hydrodynamic radius change from 12 to 37 nm) and shapes (spherical, cube, and pyramid) as a result of metal complex reduction using ascorbic acid. The process of PdNP deposition on ACF was carried out in the microreactor system. As a result, spherical-shaped PdNPs were deposited on the carbon surface. The amount of catalytic material was dependent on the used flow rate. Gong et al. [37] used the atomic layer deposition (ALD) technique for tuning spherical PdNP sizes on the AC surface. Spherical PdNPs were also deposited on activated carbon and activated carbon—the multiwalled carbon nanotube structure. Spherical—shaped PtNPs deposited on ACF as catalyst carriers [36] were also synthesized. The metallic Pt was observed on the activated carbon surface in the form of small spherical islands (D < 500 nm) containing (35 nm thick) flake-shape particles [30]. Therefore, activated carbon meets the requirements of the W4P concept.

The aim of the research was to develop an efficient and cheap method for metal ion removal from waste solution and processing of material into a catalyst (W4P approach). For this purpose, synthetic waste solutions with low content of metals (Pd and Pt) or their mixture were performed. An activated carbon, containing surface functional groups, was used as an efficient adsorbent of metal ions and also as a catalyst carrier of the final product. The process of metal adsorption was carried out in the batch reactor at different synthetic waste solution compositions, at constant metal ions concentration and temperature. As a result, the kinetic rate constants of the adsorption process were determined. Next, the obtained materials (solution after adsorption process and metal deposited on AC) were characterized using different techniques such as UV–Vis, Raman, IR spectroscopy, SEM/EDS, TGA, and XRD. The novelty of this work was obtaining a unique catalyst morphology and different (electro)catalytic properties. The collected results are a promising vision for W4P concept development for real industry samples.

## 2. Materials and Methods

### 2.1. Reagents

Metal precursors. As precursors, a base solution of H_2_PtCl_4_ (0.076 mol/dm^3^) and H_2_PdCl_4_ (0.093 mol/dm^3^) obtained from pure metals (Mennica Państwowa, 99.99%, Warsaw, Poland) were used. These solutions were prepared according to our previous work [35,38]. For adsorption tests, a freshly prepared solution containing Pt(IV) and Pd(II) ions with a concentration of 0.5 mM was used. For this purpose, a proper volume of base solution was diluted in 50 mL of deionized water. The prepared solutions were a synthetic mixture of waste solution.

Other chemicals. For analysis of waste solution generated after the adsorption process, samples were mixed in a volumetric ratio 1:1 with 0.1 mol/dm^3^ solution of HCl (p.a., Advanced Performance, Poland, obtained from concentrated solution 36–38%).

Activated carbon pellets (NORIT Activated Carbon ROY 0.8, CABOT Corporation, Cabot Aerogel GmbH, Frankfurt am Main, Germany) without further modification were used as efficient metal ions absorbers. The parameters of adsorbent included the Brunner–Emmett–Teller surface area (1062 m^2^g^−1^), V_tot_ (0.66 mLg^−1^), V_micro_ (0.53 mL g^−1^), and V_meso_ (0.13 mL g^−1^) [39].

### 2.2. Method of Analysis

UV–Vis analysis was performed using a spectrophotometer (PC—2501, Shimadzu, Kyoto, Japan) working in the wavelength range of 190–900 nm and absorbance window from 0 to 5 a.u. The collected samples during experiments were introduced to quartz cuvettes (path length 2 mm or 1 cm, Hellma, Hellma Analytics, Hellma GmbH&Co. KG, Müllheim, Germany) and then inserted into thermostat cells. As a reference, a cuvette containing solvent was used. Obtained spectra were processed using Origin 2020 software.

Raman analysis. Raman studies were carried out using Horiba Scientific Labram HR Odyssey (Horiba France SAS, Villeneuve-d’Ascq, France) equipped with a 532 nm diode laser. An 1800 diffraction grating along with the 100× Olympus objective was employed to conduct measurements. Each sample was measured as an XY map of 4 × 4 points with a step of 1 um. Laser power was adjusted to prevent sample degradation. Each map was averaged to a single spectrum, therefore giving a better S/N ratio. Spectral deconvolutions were carried out with the help of Bruker OPUS 7.2 software. RMS noise after curve fitting was below 1 for each sample.

Fourier Transform Infrared (FT-IR) analysis was performed using Bruker Vertex 70v spectrometer (Bruker, Billerica, MA, USA) equipped with Harrick Scientific Praying Mantis Diffused Reflection (DRS) attachment. Samples were measured in the 4000–400 cm^−1^ range with 4 cm^−1^ resolution. In total, 256 scans were accumulated for each measurement.

TGA analysis. TGA tests were performed in an argon atmosphere using a TA Instruments SDT Q 600 thermal analyzer. The analysis was carried out in an argon atmosphere flowing at a capacity of 100 cm^3^/min under conditions of temperature increasing at a rate of 10 deg/min. The obtained results were processed using Origin 2020 software.

SEM observations and EDS analysis. The microstructure of deposited materials was studied using an ultra-high-resolution analytical Hitachi SU-70 Schottky field emission scanning electron microscope (SEM). Observations were performed on a longitudinal and a cross-section of as-deposited samples by using secondary electrons (SE) and back-scattered electrons (BSE) imaging. The chemical composition of deposited products was confirmed by Energy Dispersive Spectrometry (EDS).

XRD analysis. The XRD method (Rigaku MiniFlex II) analyzed the phase composition using a copper tube (λ = 1.54059). Experiments were carried out in a range of 2-theta values between 10 to 80 degrees with a scan rate of 0.5 deg/min. Detailed XRD patterns were acquired with a slower scan rate of 0.1 deg/min in the range between 35 to 50 degrees. The obtained diffractograms were compared with characteristic cards from the ICDD database.

Catalytic tests. The electrochemical investigations were performed with an SP-300 BioLogic potentiostat. A platinum mesh (2 cm^2^) was used as the counter electrode, Hg/Hg_2_Cl_2_ (Sat. KCl) as the reference electrode, and different ROY-based materials as working electrodes. The ROY pellets were glued into the glassy-carbon electrode working area: 0.196 cm^−2^ by one droplet of the carbon glue and set to dry the electrode. All electrochemical experiments were performed in 1 M H_2_SO_4_ solution.

The values of potential in the presented research have been recalculated to a reversible hydrogen electrode (RHE) according to Equation (1), as follows:

(1) $$E_{RHE}=E_{Hg/{Hg}_{2}Cl_{2}}+0.059pH+{E{}^{\circ}}_{Hg/Hg_{2}{Cl}_{2}}$$

where $E_{RHE}$ is the converted potential vs. RHE, $E_{Hg/{Hg}_{2}Cl_{2}}$ is the experimentally measured potential against Hg/Hg_2_Cl_2_ reference electrode, and ${E{}^{\circ}}_{Hg/Hg_{2}{Cl}_{2}}$ is the standard potential of Hg/Hg_2_Cl_2_ at 25 °C (0.245 V).

The open circuit potential (OCP) measurements for the working electrode in the H_2_SO_4_ electrolyte were performed to establish the starting point for linear voltammetry scans. Double layer capacitance measurements were carried out in a range between 0.05 and −0.05 V vs. OCP with different scan rates. Moreover, further CV experiments were performed in a potential regime from OCP to −0.75 V vs. RHE with a scan rate of 50 mV s^−1^. Obtained scans were also presented in the logarithmic scale (−log(i) vs. E) and used to Tafel slope estimation in a current range between −10 and −1 mA cm^−2^.

## 3. Results

### 3.1. Process of Metal Recovery from Synthetic Waste Solution and Catalyst Synthesis

#### 3.1.1. Pd(II) and Pt(IV) Adsorption on Active Carbon Pellets (ROY 0.8)—Kinetic Study

**Figure 1:**
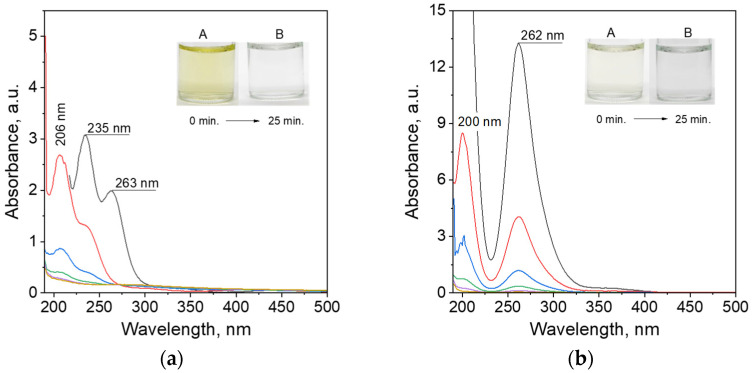

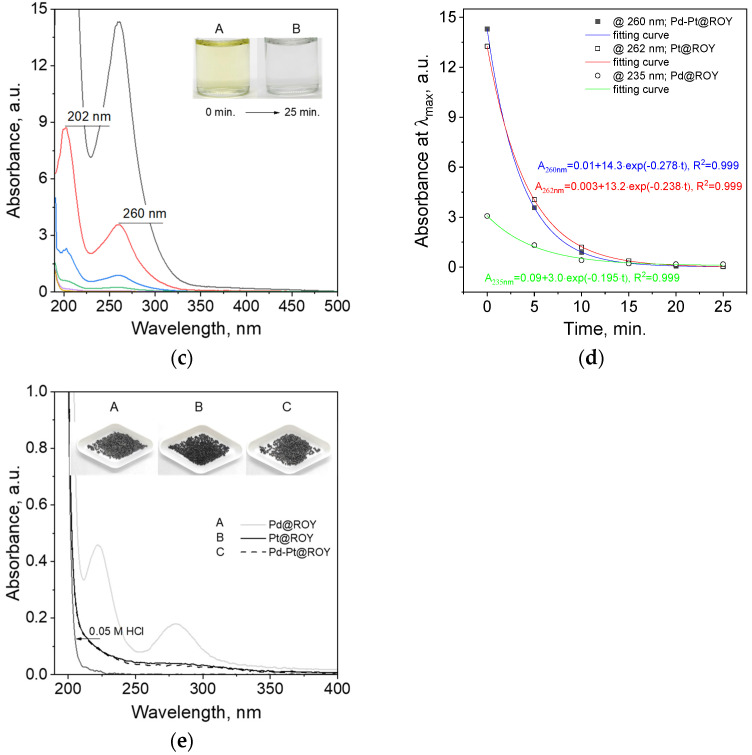
Colors of the samples and spectra evolution for Pd@ROY (**a**), Pt@ROY (**b**), and Pd-Pt@ROY (**c**) obtained during the adsorption process (time of analysis: black—0, red—5, blue—10, green—15, purple—20, and brown—25 min.); kinetics curves at selected λ_max_ with fittings obtained for Pd@ROY, Pt@ROY, and Pd-Pt@ROY (**d**); filtrate analysis and carbon pellets after the adsorption process, A—Pd@ROY; B—Pt@ROY; and C—Pd-Pt@ROY (**e**). Conditions: m_ROY_ = 0.5 g, C_0, Pd(II), Pt(IV)_ = 5 × 10^−4^ mol/dm^3^, V = 50 mL, T = 20 °C, 500 rpm, time = 25 min, and interval = 5 min. Note: the values of absorbance were recalculated from path length 0.2 cm to 1 cm (results shown in (**b**–**d**)).

The process of metal ions adsorption on activated carbon (ROY 0.8) was carried out in the batch reactor (glass bottle, Duran) with a volume of 100 mL and a mixing rate of 500 rpm (3 cm long mixing bar with diameter: 0.8 mm). During the process, the 2.5 mL of solution was collected and analyzed using spectrophotometry. After analysis, the solution was returned to the batch reactor. Obtained spectra for different samples, i.e., Pd@ROY, Pt@ROY, and Pd-Pt@ROY, were shown in Figure 1a–c.

Registered spectra of Pd(II) and Pt(IV) ions have characteristic peaks at different wavelengths. For the Pd(II) aqueous solution, there are 206, 235, and 263 nm (Figure 1a). Whereas, for Pt(IV) ions, the spectrum with a maximum wavelength at 200 and 262 nm (Figure 1b) was registered. The mixture of both metal ions has summarized the spectrum with maxima at 202 and 260 nm (Figure 1c). After mixing a solution containing metal ions with activated carbon, the change in color from yellow coming from Pd(II) and Pt(IV) to colorless was observed (Figure 1a–c). The obtained spectra evolution (Figure 1a–c) confirmed adsorption of metal ions on carbon pellets. Based on these spectra, at selected wavelengths, the kinetic curves were drawn (Figure 1d). In each case, the curve has an exponential character suggesting that the process is first order with respect to metal ions (M_i_). According to Wojnicki et al. [30], the process of adsorption of metal ions on activated carbon can be written as follows:

(2) $$M_{i}+AC\overset{k_{a}}{\to }M_{i}@AC$$

where $M_{i}$—metal ions; AC—activated carbon, ROY; $M_{i}@AC$—metal ions adsorbed on AC surface; and $k_{a}$—rate constant.

For this reaction, the differential equation describing the change in the metal ions concentration during adsorption process has the following form:

(3) $$\frac{d[M_{i}]}{dt}=-k_{a}\cdot [AC]\cdot [M_{i}]$$

Taking into account a great amount of carbon comparing to metal ions, the process is pseud-first order and the observed rate constant ($k_{obs})$ can be written as:

(4) $$k_{obs}=k_{a}\cdot [AC]$$

The Equation (3) takes the following form:

(5) $$\frac{d[M_{i}]}{dt}=-k_{obs}\cdot [M_{i}]$$

The solution of the differential Equation (5) takes the following form:

(6) $${[M}_{i}]=[M_{i}{]}_{t\to \infty }+{[M_{i}{]}_{0}\cdot e}^{-k_{obs}t}$$

where $[M_{i}{]}_{0}—$initial concentration of metal ions in the solution; $[M_{i}{]}_{t\to \infty }$—concentration of metal ions at the end of the adsorption process; and ${[M}_{i}]$—metal ions concertation at time “t”. According to the Lambert–Beer law, absorbance is proportional to the concentration of metal ions (A $\propto [M_{i}]$).

The Equation (6) had the same formula as the fitted equation to experimental data (Figure 1d). It confirmed that the process of metal ions adsorption on activated carbon is first order. Taking into account the large amount of used carbon compared to metal ions, the process is pseudo-first order. From exponential fit to kinetic data, the value of observed rate constant was determined and gathered in Table 1.

Comparing these data, it can be seen that the process runs faster in the solution containing a mixture of Pd(II) and Pt(IV) ions. The fact that the rate of adsorption process for Pt(IV) ions is faster than for Pd(II) ions is also interesting. Taking into account that the process of adsorption was carried out at pH = 3, it can be concluded that in the solution, different complexes coexist. These forms adsorb at different rates. Among them, in the case of Pd(II) ions, coexists [PdCl_4_]^2−^ and [PdCl_3_(H_2_O)]^−^ formed in reaction (7). Depending on the solution composition and sorbent used in adsorption process, different Pd(II) species are preferable. Nakano et al. [40] showed that [PdCl_2_(H_2_O)_2_] and [PdCl(H_2_O)_3_]^+^ complexes adsorb faster than [PdCl_3_(H_2_O)]^−^ and [PdCl_4_]^2−^. In this process, condensed-tannin gel particles were used for the adsorption process. Whereas, Wojnicki et al. [28] indicated that in acidic solution containing chloride ions, the [PdCl_4_]^2−^ complex adsorbs on the activated carbon (GF40 Norit) surface.

(7) $${[PdCl}_{4}{]}^{2-}+H_{2}O\overset{K_{1a}}{↔}{[PdCl}_{3}(H_{2}O){]}^{-}+{Cl}^{-}$$

The hydrolysis of Pd(II) is a fast process (logK_1a_ = 5.08 at 20 °C) [41]. The released chloride ions (Equation (7)) also adsorb on the carbon surface [42]. This fact leads to a shift of equilibrium toward products. However, ${[PdCl}_{4}{]}^{2-}$ can be also adsorb on carbon surface [43]. Thus, these processes are competitive. Undoubtedly, the recorded spectra (Figure 1a) indicate the presence of a hydrolyzed form of palladium. Thus, registration of spectra after the adsorption process was possible only after hydrochloric acid addition. Considering the weak acidic conditions (pH = 3), the most probable is the further hydrolysis (8–10), according to [41].

(8) $${[PdCl}_{3}(H_{2}O){]}^{-}+H_{2}O\overset{K_{2a}}{↔}{[PdCl}_{2}(H_{2}O{)}_{2}{]}_{cis}+{Cl}^{-}$$

(9) $${[PdCl}_{3}(H_{2}O){]}^{-}+H_{2}O\overset{K_{3a}}{↔}{[PdCl}_{2}(H_{2}O{)}_{2}{]}_{cis/trans}+{Cl}^{-}$$

(10) $${[PdCl}_{2}(H_{2}O{)}_{2}{]}_{cis/trans}+H_{2}O\overset{K_{4a}}{↔}{[PdCl}_{2}(H_{2}O{)}_{2}{]}^{+}+{Cl}^{-}$$

For reactions (8–10), the values of stability constants log K_2a–4a_ equal 3.475, 2.1, and 0.902, respectively.

Pt(IV) ions also undergo hydrolysis process. Selected species and reactions were listed below, considering pH < 7 (11–13), as follows:

(11) $$[Pt{Cl}_{6}{]}^{2-}+H_{2}O\overset{K_{1b}}{↔}[Pt{Cl}_{5}\left(H_{2}0\right){]}^{-}+{Cl}^{-}$$

(12) $$[Pt{Cl}_{5}\left(H_{2}0\right){]}^{-}+H_{2}O\overset{K_{2b}}{↔}[Pt{Cl}_{4}({H_{2}0)}_{2}]+{Cl}^{-}$$

(13) $$[Pt{Cl}_{4}(H_{2}O{)}_{2}]+H_{2}O\overset{K_{3b}}{↔}[Pt{Cl}_{3}({H_{2}0)}_{2}{]}^{+}+{Cl}^{-}$$

where K_1b_, K_2b_, and K_3b_ are the equilibrium constant of reactions (11)–(13), respectively. For these reactions, values of equilibrium constant equal log K_1b_ = 1.5–2.2, log K_2b_ = 3.7, and log K_3b_ = 3.5 [44].

Taking into account the pH of the solution and the small amount of chloride ions, during the adsorption process, we have 100% of the $[Pt{Cl}_{5}\left(H_{2}0\right){]}^{-}$. This is in accordance with the literature data [44].

Acceleration of the adsorption rate in the case of the binary system (Pd(II) and Pt(IV) ions) leads to the conclusion that the sum of the chloride ions in the solution is higher than in other studied systems. Therefore, there are more non-hydrolyzed species in the bimetallic system. These species adsorb faster on the activated carbon surface compared to single metal adsorption.

After the adsorption process, solid samples (ROY with adsorbed material) were separated from the solution via the filtration process. Next, solid samples were dried for 1 h at 70 °C and left to cool down at temperature 20 °C. Depending on the adsorbed metal, colors of the samples changed from black for ROY to grey for Pd@ROY (Figure 1, Sample A) and Pd-Pt@ROY (Figure 1, Sample C). However, Pt@ROY did not change color after the adsorption process (Figure 1, Sample B). It suggests that palladium ions probably adsorbed and reduced on the carbon surface, whereas platinum ions had to penetrate deep into the material and that is why we do not see any changes on the surface. In order to confirm our supposition, solid samples were analyzed using different techniques (see next Section 3.1.2, Section 3.1.3, Section 3.1.4, Section 3.1.5, Section 3.1.6 and Section 3.1.7). Moreover, the waste solution generated after the adsorption process was also analyzed but after its previous mixing with 0.1 mol/dm^3^ solution of HCl (volumetric ratio 1:1). The addition of HCl to the filtrate solution suppresses and reverses the hydrolysis process [45]. Thanks to this, the UV–Vis spectra can be registered. The obtained results are shown in Figure 1e. The UV–Vis spectrum was also analyzed for 0.05 mol/dm^3^ solution of HCl. In all of the analyzed samples, below 220 nm, a strong increase in the spectrum intensity was recorded. It comes from hydrochloric acid, which absorbs strongly below 210 nm (as a reference sample water was used) [46]. It was also confirmed by registration of the spectrum for 0.05 M HCl solution, shown in Figure 1e. Only the spectrum with a maximum at c.a. 220 and 280 nm, for the waste solution after the process of Pd(II) ion adsorption on ROY (Figure 1e), was recorded. The location of these peaks is consistent with peak locations typical for Pd(II) ions in the form of the ${[PdCl}_{4}{]}^{2-}$ complex, expected in such pH conditions [41,47]. The amount of Pd(II) ions remaining in the filtrate is 1.5% of the initial value. Analysis of the solution after process (samples Pt@ROY and Pd-Pt@ROY) showed that some compounds were released from the AC surface. The location of the band in the range of the wavelengths 220–350 nm (see, Figure 1e) implies that this absorption signal might come from groups released/desorbed from activated carbon surface and may belong to carboxylic [48,49] and phenolic compounds [50].

Moreover, we carried out the process of ROY impregnation in a blank solution containing only a small amount of chloride ions (the amount of chloride ions was similar to that introduced to the solution together with metal precursors for adsorption studies). The obtained spectra during the adsorption process are shown in Supplementary Materials, Figure S1. Obtained spectra evolution confirmed the adsorption of chloride ions on the ROY surface. This may also suggest that the further adsorption process of metallic species takes place via the chloride link.

#### 3.1.2. Raman Analysis of ROY, Pd@ROY, Pt@ROY, and Pd-Pt@ROY

**Figure 2:**
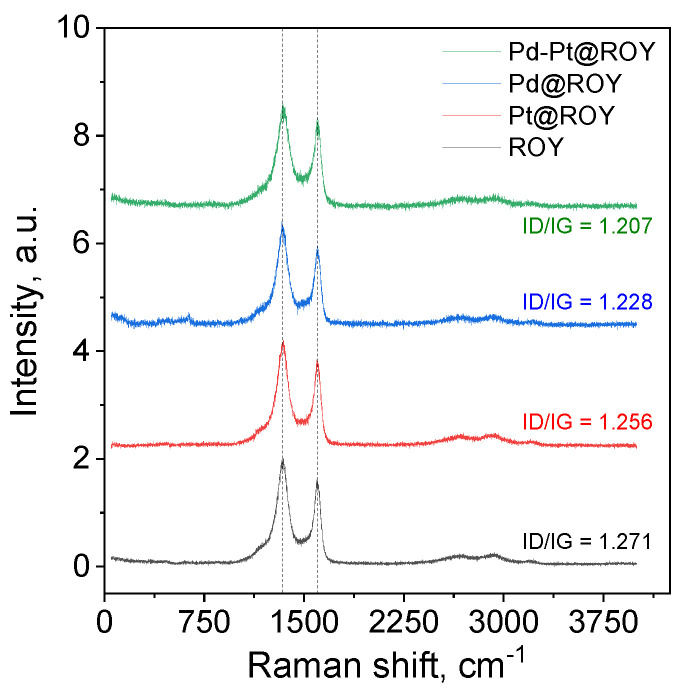
Raman spectra for ROY, Pd@ROY, Pt@ROY, and Pd-Pt@ROY samples.

Presented Raman spectra (Figure 2) are typical for activated carbons [51,52,53], as indicated by the presence of the D (approx. 1343 cm^−1^) and G bands (approx. 1600 cm^−1^) and combination bands of very low intensity—2D (approx. 2650 cm ^−1^), D+G at 2930 cm^−1^, and 2G at 3200 cm^−1^ [51,52,53,54]. A careful analysis of the spectra shows that there are no visible changes in the combination bands’ positions and the intensity changes after the addition of metals are very small. No chlorides or oxides were detected, thus suggesting that adsorbed metals should be at zero oxidation state. It implies that adsorbed metal ions were reduced by surface groups to the metallic state.

In order to conduct a more precise analysis of the obtained materials and the influence of catalysts on the supports, the range of 1000–1800 cm^−1^ was subjected to the process of decomposition into component bands. The output data are presented in Table S1 (Supplementary Materials). As a result, a set of bands was obtained and example fits are presented in Figure S2 (Supplementary Materials). The 1170 cm^−1^ band can be probably assigned to the mixed sp^2^–sp^3^ disordered carbons [54]. The 1360 and 1600 cm^−1^ bands are typical D and G bands—the A_1g_ breathing mode of hexagonal rings and E_2g_ symmetry in-plane stretching LO phonons of the Brillouin zone, respectively [51,52,53,54]. Also, an additional pair of bands is observed, at 1300 and 1550 cm^−1^, which could be named D2 and G2. They originate from the amorphous carbon leftover from the activation process with bond angle disorder [51,52]. Their presence is common for the activated carbon samples [51]. Based on the obtained results, the I_D_/I_G_ intensity ratio was calculated as shown in Table 2.

The measured intensity of the D and G bands ratio decreases in the order ROY > Pt@ROY > Pd@ROY > Pd-Pt@ROY. Registered changes are very subtle, as shown in Table 2, but it means that a higher level of structural disorder/defects on carbon-based material was observed for ROY [51]. This implies that the presence of Pt and/or Pd contributes to the changes in the surface of the material. The lowest value of the I_D_ to I_G_ ratio was calculated for Pd-Pt@ROY, suggesting a more ordered structure. At the same time, assuming that the G2 band comes from the amorphous carbon phase, it is clear that the addition of metal also affects the behavior of this band, which is evidenced by the change in the intensity. The calculations of the I_D2_/I_G2_ ratios confirm the observed trend for the main bands as presented in Table S1 (Supplementary Materials). This means that the metal particles change the surface of carbon materials in a very subtle way.

For the calculation of the average lateral extension of the graphene planes, the Cançado et al. [55] approach has been used (Tuinstra Koenig Relation), Equation (14).

(14) $$L_{a}=(2.4\times 10^{-10})\times \lambda^{4}\times (I_{D}/I_{G}{)}^{-1}$$

As presented in Table S1 (Supplementary Materials), the $L_{a}$ parameters change according to the intensity ratio changes for both G and D as well as the G2 and D2 bands. The changes for the main bands are very small and it seems that the metal only slightly affects the change in the crystallinity of the materials surface. This is consistent with the general picture of the recorded spectra, where the changes, if visible, are very small. In the case of the D2 and G2 bands, the change is much more visible, which may indicate that the metal is located in the amorphous carbon/more highly defected structure region. Its influence in this case is very clear.

#### 3.1.3. IR Analysis

**Figure 3:**
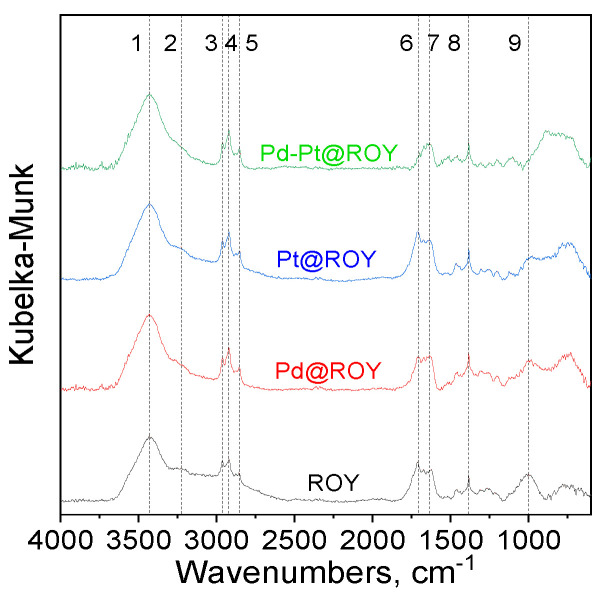
IR analysis performed for ROY, Pd@ROY, Pt@ROY, and Pd-Pt@ROY samples and selected bands. Notation: 1—3430; 2—3230; 3—2961; 4—2914; 5—2851; 6—1710; 7—1640; 8—1385; and 9—1000 cm^−1^.

The performed FT-IR measurements showed that all of the tested samples are similar to each other but with different peaks intensity (Figure 3 and Supplementary Materials, Figure S3).

In each case, there is a clear band at approximately 3430 cm^−1^ coming from adsorbed water (vas O-H) [56]. This is also confirmed by the band at approximately 1640 cm^−1^ (H-OH), which may also originate from C=C bonds in aromatic compounds [56,57,58]. A band at approximately 3230 cm^−1^ is also visible, which can most likely be associated with the presence of carboxylic acid formed as a result of carbon activation [56]. These bands decrease in intensity for each of the added metals and the most for the Pd-Pt mixture. This indicates that these bonds are involved in the immobilization of the above-mentioned compounds. For each material, bands typical of C-H vibrations are also visible at approximately 2900 cm^−1^ (for both asymmetric and symmetric stretching vibrations) [56,57,58]. The bands at approximately 1740 and 1710 cm^−1^ come from C=O bonds—the weaker band at higher wavenumbers probably comes from the ester group, while the other one comes from the carboxylic acid [58,59,60]. With the addition of Pt, these bands basically do not change but the addition of Pd causes a reduction in their intensity. For the Pd-Pt@ROY spectrum, the band at approximately 1740 cm^−1^ almost disappears and a very significant weakening of the intensity of the 1701 cm^−1^ band is observed. One can also see, for each of the tested materials, a shift of the C=O bands toward lower wavenumbers. This directly indicates that the immobilization reaction takes place at these centers. The band at approximately 1460 cm^−1^ can be assigned to both C-H deformation vibrations and C-O vibrations in carboxylic acid, which is also reflected in the disappearance of this band with the addition of Pd-Pt. Each of the spectra has a band at approximately 1385 cm^−1^, which indicates activation with nitric acid, as this is a typical band for N-O bonds in NO_3_ units. The bands in the range of 1300–1000 cm^−1^ come from C-O and C-O-C bonds [56,57,58]. The broad bands that are visible at approx. 1000 cm^−1^ and around 800–700 cm^−1^ are most probably leftovers from the activation process. These observations for the Pt, Pd, and Pd-Pt samples are in good agreement with Raman data, where the largest change in the I_D_/I_G_ ratio is observed for the Pd-Pt@ROY sample.

#### 3.1.4. Thermal Gravimetric Analysis of the Obtained Catalyst

**Figure 4:**
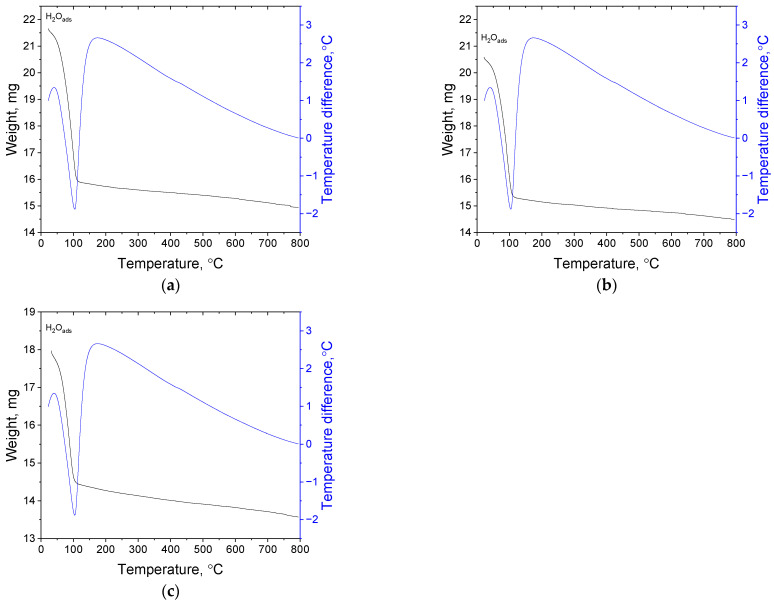
TGA of Pd@ROY (**a**); Pt@ROY (**b**); and Pd-Pt@ROY (**c**). Notation: $H_{2}O_{ads}$—adsorbed water.

To detect the formula of possible compounds adsorbed on the ROY surface, TGA analysis was carried out. The change in the mass during material heating up to 800 °C was registered under argon conditions. In all of the samples, a large decrease in mass up to 110 °C was observed (see, Figure 4). These mass changes came from adsorbed water. The presence of a strong endothermic peak with a maximum of 100 °C also confirmed this.

The percentage amount of released water under sample heating (up to 110 °C) equals 25.9% for Pd@ROY, 25.4% for Pt@ROY, and 19.4% for Pd-Pt@ROY. Further sample heating (up to 800 °C) most probably released the compounds present on the activated carbon surface. There are carboxylic and phenolic groups, which, with increasing temperature, can be transformed into anhydride or lactone groups [61]. These compounds release CO_2_ and CO at about 450 °C. The DTA curve should indicate some visible exothermic peak near this temperature. On the first look (example, Figure 4a), we are unable to detect any thermic effect. However, in the temperature range 400–450 °C, some “a bulge” can be seen in the graph (see, Supplementary Materials, Figure S4a). To prove it, derivatives of temperature were calculated and the obtained result is shown in Figure S4b (Supplementary Materials). This allowed us to visualize the exothermic effect, which, in the case of sample Pd@ROY, had its maximum at 425 °C. The location of this peak is 25 °C far from that reported in the literature. However, such a shift might relate to carbon material. Summarizing obtained data from TGA analysis, the percentage mass changes in temperature range 110–800 °C equals 6.7% for Pd@ROY, 5.5% for Pt@ROY, and 6.6% for Pd-Pt@ROY. For sample Pt@ROY, the amount of surface groups is the smallest (see, Supplementary Materials, Figure S4b). It might suggest that during the adsorption process, platinum ions react with these groups in all volumes of the carbon pellet. However, this was not observed for the Pd-Pt@ROY sample (see, Supplementary Materials, Figure S4d), in which mass change differed by only 0.1% from the Pd@ROY sample. It might suggest that in the case of sample Pd-Pt@ROY, another mechanism should be considered compared to the Pt@ROY sample. In order to check this, all samples were analyzed using SEM (see, Section 3.1.5).

#### 3.1.5. SEM/EDS Analysis

**Figure 5:**
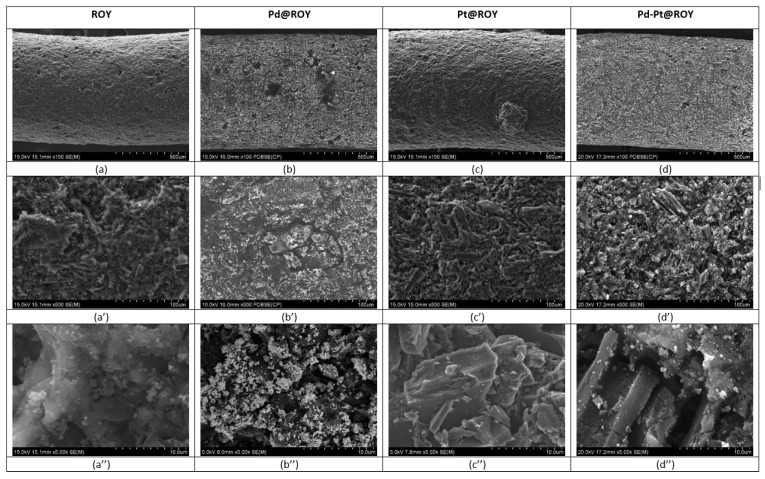
SEM analysis of ROY (**a**–**a’’**), Pd@ROY (**b**–**b’’**), Pt@ROY (**c**–**c’’**), and Pd-Pt@ROY (**d**–**d’’**) samples at different magnifications.

The morphology (surface and cross-section of the sample) of deposited products was analyzed using SEM. The obtained results for selected samples at different magnifications are shown in Figure 5 and in Supplementary Materials, Figure S5.

Different sample magnifications used during SEM observations enable a thorough analysis of the materials. When comparing samples ROY and Pt@ROY, their outer surfaces appear similar (Figure 5a,c). SEM analysis of the Pt@ROY sample did not reveal any nanoparticles deposited on the carbon surface (Figure 5c–c’’). EDS analysis of the sample surface (Supplementary Materials, Figure S6, Table S2) detects a low amount of platinum. A cross-section of the sample and EDS analysis at the selected area (Supplementary Materials, Figure S7) indicates 11% (weight) platinum (Supplementary Materials, Table S3). It confirmed that platinum ions had to penetrate the pores of activated carbon.

**Figure 6:**
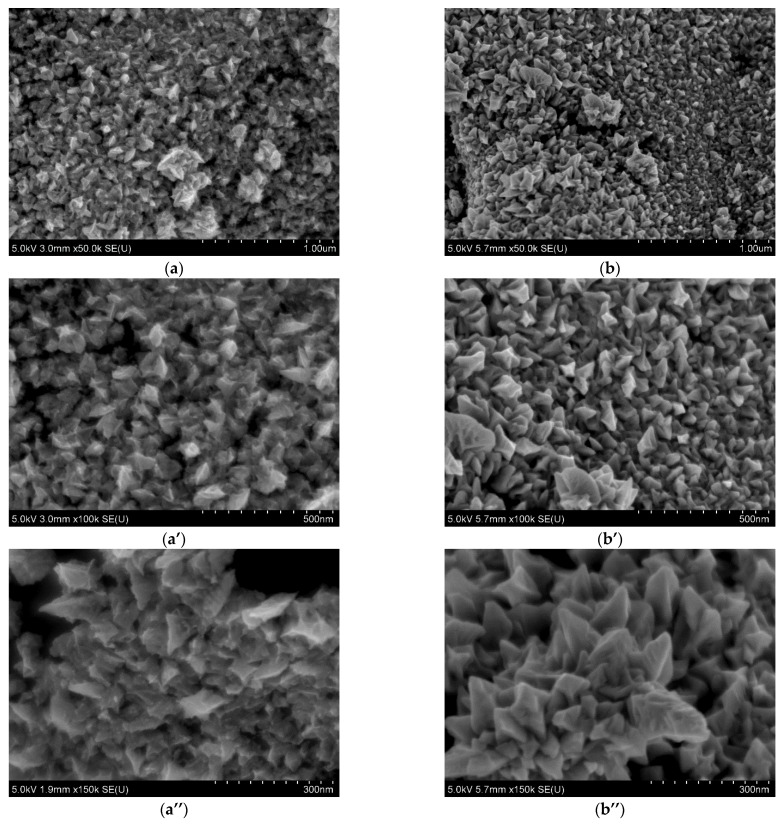
Detailed SEM analysis performed for Pd-Pt@ROY (**a**–**a’’**) and Pd@ROY_Cl (**b**–**b’’**) at different magnifications.

The most interesting observations were noted for the Pd@ROY and Pd-Pt@ROY samples (Figure 5 b–b’’,d–d’’, Supplementary Materials, Figure S8). While their outer surfaces look similar at lower magnifications (Figure 5b,d), higher magnifications revealed significant differences between them. The Pd@ROY surface was heavily covered by metallic palladium (around 72%, weight), as confirmed by EDS chemical analysis (see, Supplementary Materials, Figure S9, Table S4). As it was expected, the cross-section of the sample, obtained after its breaking, did not show a signal from palladium (see, Supplementary Materials, Figure S10, Table S5). This suggests that Pd(II) ions are first absorbed and then reduced to the metal by functional groups formed during carbon activation. Both small palladium particles (50 nm) and large dendrites growing in larger colonies were observed on the carbon surface (Supplementary Materials, Figure S8), wherein these deposits only occupied the outer surface of the carbon granule. In contrast, the Pd-Pt@ROY sample develops a slightly different microstructure of the deposits (Figure 6a–a’’). Higher magnification observations revealed fine-scale deposits coexisting with a small number of coarse-grained aggregates. As a result of Pd and Pt adsorption on ROY, a pyramid-like structure on the carbon surface was observed (Figure 6a–a’’). EDS analysis suggested the presence of both metals incorporated in the pyramid structure (see, Supplementary Materials, Figures S11 and S12, Tables S6 and S7). The amount of platinum was less than that of palladium, suggesting that some platinum ions might penetrate deeper into the carbon structure or were too small to be detected at the magnification regime offered by the SEM. Analysis of the sample cross-section indicates only a small amount of both metals (Supplementary Materials, Figure S13, Table S8). It suggests other adsorption mechanisms compared to the sample Pt@ROY. Most probable is the coexistence of galvanic replacement, which results in the reduction of platinum ions.

The presence of a pyramid structure containing palladium is an interesting observation that has not been documented in the literature so far. Skibińska et al. [62] observed that the shape of metallic nanocones depends on the concentration of ions, including chloride. In order to confirm the role of the chloride ions in the process of nanopyramid growth, we made additional experiments. The lowest amount of chloride ions was set in such a way that its amount was similar to the composition of the Pd-Pt@ROY sample and equals c.a. 3 mmol/dm^3^. The morphology of both samples Pd-Pt@ROY and Pd@ROY_Cl were compared in Figure 6.

The obtained results confirmed that chloride ions promote palladium nanopyramid formation (Figure 6). Moreover, the addition of Pt(IV) ions during the adsorption process caused the incorporation of platinum nanoparticles (see, Figure 6a’’) into the pyramid structure. More details with marked PtNPs on SEM analysis are shown in Supplementary Materials (Figure S14). The EDS analysis of the composition along the nanopyramide (Pd-Pt@ROY) structure (Supplementary Materials, Figures S15 and S16) suggests that the structure contains both palladium and platinum. Moreover, EDS analysis along the nanopyramid structure indicated a change in Pd and carbon amount, whereas the amount of Pt was constant (see, Supplementary Materials, Figures S15b and S16b). In order to more precisely characterize this material or to confirm the distribution of Pd and Pt in the structure, more precision microscopy should be used. However, the application of higher magnification during SEM analysis allowed us to distinguish brighter points (platinum is a heavier metal than palladium), which stands out against the pyramidal structure (see, Figure 6a’’, Supplementary Materials, Figure S14). In addition, TGA analysis was performed for sample Pd@ROY_Cl and compared to pure ROY containing only the addition of chloride ions during the adsorption process (see, Supplementary Materials, Figure S17a,b). The percentage amount of released water under sample heating (up to 110 °C) equals 3.1% for Pd@ROY_Cl and 5.3% for ROY_Cl. The amount of adsorbed water for these samples is much lower than that obtained for other studied materials (see, Section 3.1.4). Most probably, it results from the change in the wettability of the surface due to a change in surface morphology (see, Section 3.1.6). Further sample heating (up to 800 °C) causes the release of functional groups (carboxylic and phenolic) present on the ROY surface. The percentage mass changes equal 5.8% for Pd@ROY_Cl and 5.6% for ROY_Cl and are similar to the value obtained for the Pt@ROY sample (see, Section 3.1.4). Additional analysis (Supplementary Materials, Figure S17c,d) allowed broad exothermic peak detection between 390 and 450 °C. These results might suggest some interaction between the carboxylic group and chloride ions [42]. To confirm this, more detailed data focusing on this issue need to be obtained.

#### 3.1.6. Wettability Tests

Due to the small size of used carbon pellets, it was not possible to make tests dedicated to wettability measurements. Thus, other tests were performed. Two samples containing eight pieces of carbon (Pd@ROY and Pd-Pt@ROY) with a total mass of 5.5 mg each were introduced at once to two batch reactors containing 3 mL of water. Immediately, in the reactor containing Pd@ROY, the gaseous bubbles on the carbon surface were observed. Whereas, in the second batch reactor containing Pd-Pt@ROY, bubbles appeared a few seconds later. These experiments confirmed that wettability is larger in the case of Pd@ROY and gas trapped inside of carbon pore is released faster than for the Pd-Pt@ROY sample. This test also implied that the pyramid-like structure is characterized by greater hydrophobicity and it is in accordance with the literature data related to similarity in the shaped structure [63].

#### 3.1.7. XRD Analysis

**Figure 7:**
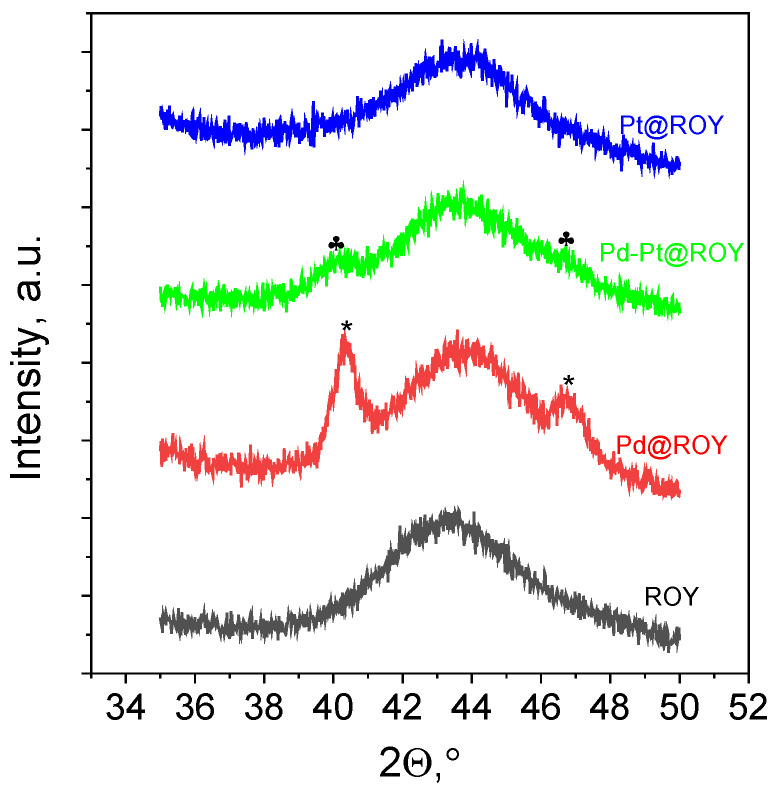
The fragments of XRD patterns with peaks assignments * for Pd@ROY: 40.333 and 46.855° and ♣ Pd-Pt@ROY: 40.042 and 46.621°.

For selected samples, i.e., ROY, Pd@ROY, Pd-Pt@ROY and Pt@ROY, additional XRD analyses were performed. The obtained results over the entire angular range (10–80°) were presented in Supplementary Materials, Figure S18a. A broad peak with a maximum around 22 ° registered on the XRD pattern (Figure S18a, Supplementary Materials) is close to that described in the literature [64]. This reflection comes from the (0 0 2) plane of carbon support and does not interfere with the diffraction peaks of Pd and Pt. However, a second broad peak located at 43.52° coming from (1 0 0) planes [64] partly overlaps the signal from metals (Figure 7). Deposition of metals on carbon structure leads to the shift of the peak from 22 to 23.5° (Supplementary Materials, Figure S18a), it was difficult to find a similar relation at second peak, due to peaks from support and metals overlapping (Figure 7). During analyses, no visible changes on XRD patterns were observed for Pd-Pt@ROY and Pt@ROY samples. Thus, the analyses were carried out in the selected angular range (35–50°) and greater accuracy (see, Section 2.1). Obtained XRD patterns were compared in Figure 7.

XRD peaks of (1 1 1) and (2 0 0) planes with data from the ICDD cards and the literature were gathered in Table 3. Peak assignments for Pd@ROY and Pd-Pt@ROY samples gathered in Table 3 were determined after peak deconvolution using Gaussian mode in Origin software (Supplementary Materials, Figure S18b,c).

The collected data (Table 3) confirmed the presence of palladium in the Pd@ROY sample and the small difference in peak position can refer to the carbon support, as was shown in the case of activated carbon [4]. The peak location for the Pd-Pt@ROY sample is similar to the bimetallic catalyst deposited on the ACF surface [4]. Moreover, the location of the XRD peaks of both planes is between the values for pure metals (Table 3). Unfortunately, it was not possible to record the XRD pattern for the Pt@ROY sample. This is probably due to the very small amount of metal on the surface. Additionally, a wide signal from the support has a negative impact on the detection of signal coming from platinum.

## 4. Electrocatalytic Properties of Obtained Materials for the Hydrogen Evolution Reaction

**Figure 8:**
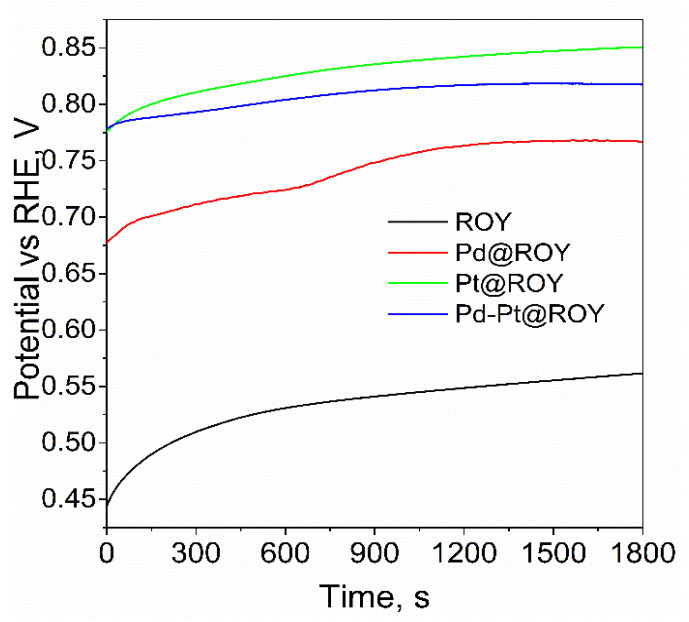
OCP measurement for ROY, Pd@ROY, Pt@ROY, and Pd-Pt@ROY samples in 1 mol/dm^3^ H_2_SO_4_ solution.

The OCP measurements of freshly prepared electrodes with bulk and modified ROY materials are presented in Figure 8.

**Figure 9:**
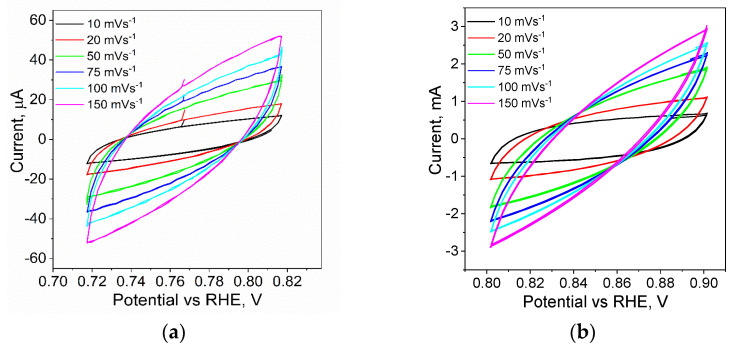
Cyclic voltammetry in 1 mol/dm^3^ H_2_SO_4_ solution for Pd@ROY (**a**) and Pt@ROY (**b**) samples as a function of scan rate.

Due to the character of the test material, which is activated carbon before and after the modification process, it was necessary to measure the potential value as a function of time. This is due to the fact that the electrolyte needed a certain amount of time to penetrate the porous structure of the carbon. For each of the samples tested, the evolution of the OCP potential value over time was observed. It was observed that the samples reached a stable state after about 15 min, which may indicate the completion of capillary penetration of the solution into the nano- and microchannels. It is also worth mentioning that the potential values for the different materials vary. Pure carbon showed an OCP potential value of −0.55 V after 20 min. A value of −0.75 V was recorded for the palladium-modified material (Pd@ROY) and −0.85 V for platinum (Pt@ROY). An intermediate potential value of −0.79 V was obtained for the Pd-Pt alloyed material. The measured values in the OCP measurements served as a starting point for measuring the double-layer capacitance in cyclic voltammetry measurements with a variable scan rate. Example curves for Pd@ROY and Pt@ROY are shown in Figure 9.

**Figure 10:**
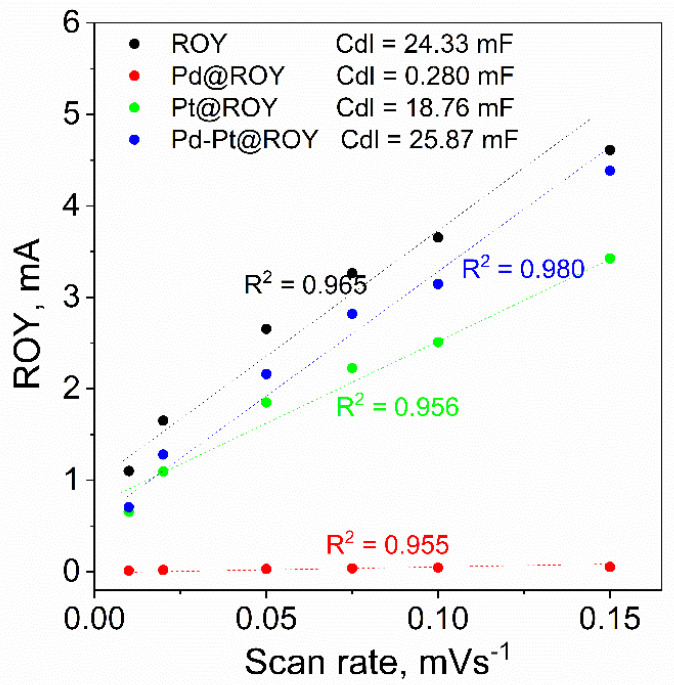
Dependence of recorded current values as a function of scanning speed for ROY, Pd@ROY, Pt@ROY, and Pd-Pt@ROY materials.

The recorded set of voltammetric curves for Pd@ROY indicates a small value of current in the selected potential range. Changing the scan rate increases the recorded signals, which is consistent with theory; however, the values are more than an order of magnitude smaller for Pd@ROY than for Pt@ROY. The capacitance value is derived from the degree of surface development, which, in the case of electron microscopy analyses, showed that in the case of Pd@ROY, we are dealing with a compact Pd shell on the surface of the activated carbon, which prevents the electrolyte from penetrating into the material. In the case of Pt and the Pd-Pt alloy, the surface morphology of the carbons looked completely different, being much more extended and heterogeneous, which is reflected in the recorded current values in cyclic voltammetry scans. The resulting sets of curves for the unmodified material as well as Pd, Pt, and Pd-Pt were plotted on a scan rate and current dependence graph to estimate the capacity of the double layer. The results are shown in Figure 10.

**Figure 11:**
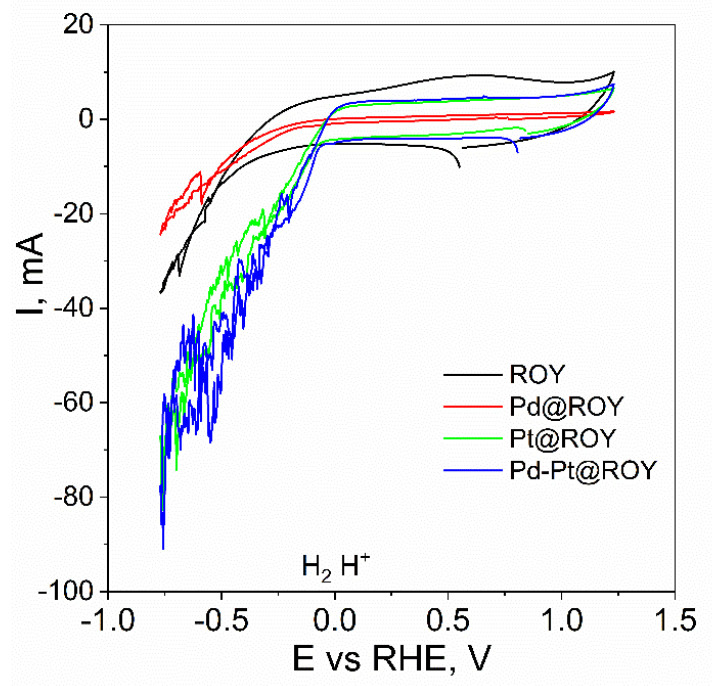
Cyclic voltammetry for ROY, Pd@ROY, Pt@ROY, and Pd-Pt@ROY materials in the potential range −0.7 V to +1.1 V.

The values of the linear function coefficient are an indicator of the capacity of the double layer of the investigated material. Carbon without modification is characterized by a very high degree of surface development with a capacitance of 24.33 mF. It should be noted that the modification of Pt carbon decreases the recorded capacitance, which may be related to the partial blocking of channels within the porous structure. SEM observations carried out for the Pd-Pt alloy material showed the formation of a developed irregular structure on the surface, which contributes to a slight increase in the recorded capacitance of the double layer. In the case of Pd@ROY, the formation of a tight palladium layer on the carbon surface, which prevents the penetration of the electrolyte into the interior of the porous material, significantly affects its electrochemical capacity, which is two orders of magnitude lower than for the material without modification. The next step in the characterization of the electrochemical properties of the tested materials was the measurement of cyclic voltammetry in a sulfuric acid solution. The curves obtained are shown in Figure 11.

The scan carried out for the unmodified ROY material shows high recorded current values in the range from 0 to +1.1 V where no electrochemical reactions are observed, indicating a high degree of surface development. Analogous observations were also made for the Pt@ROY and Pd-Pt@ROY material, which is in agreement with the results obtained from the previously realized voltammetric curves with a variable scan rate. In the case of the Pd@ROY sample, where two orders of magnitude lower double layer capacitance were recorded in the non-faraday range, the current values are small and in agreement with previous electrochemical measurements. When scanning in the range where hydrogen evolution is expected, the lowest current value was recorded for Pd@ROY, which reached about 20 mA. Interestingly, the pure carbon material showed higher catalytic activity and, at a potential of −0.7 V, the recorded current was higher than for Pd@ROY, at −38 mA. The difference in activity, according to us, is related to the almost 100-fold higher active surface area for the unmodified carbon compared to Pd@ROY, where a tight electrolyte-impermeable metallic layer formed on its surface.

**Figure 12:**
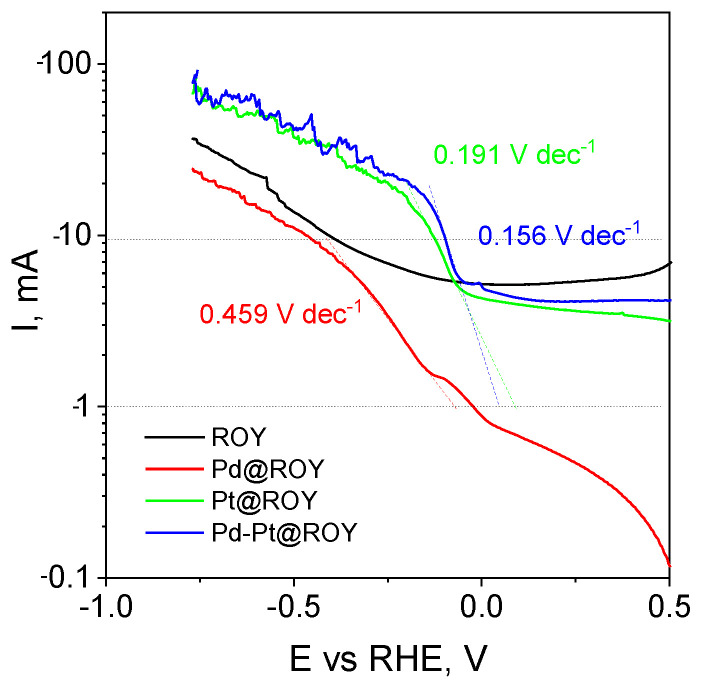
Semi-logarithmic curve of current as a function of potential with the slope of the Tafel curve plotted in the range from −1 to −10 mA ROY, Pd@ROY, Pt@ROY, and Pd-Pt@ROY.

Interestingly, the two materials also differ in the value of the potential for which the curve collapse associated with hydrogen evolution is observed, where it is −0.39 V for the pure material and only −0.25 V for Pd@ROY. The best results were obtained for the Pd-Pt@ROY alloy material, for which the maximum current at a potential of −0.7 V was −90 mA. In the case of Pt@ROY, the value was very similar at approximately −75 mA. It should be noted that the course of these curves and the point at which the hydrogen evolution reaction starts are practically identical (for Pt −0.052 V and Pd-Pt −0.041 V), which indicates a very low overpotential of hydrogen evolution on the electrodes studied. The obtained curves are presented in semi-logarithmic coordinates, which allowed the Tafel slope to be determined in the current range from −1 to −10 mA (Figure 12).

For the Pd-modified carbon material, the slope of the curve was 459 mV per current decade, indicating a strong inhibition of the hydrogen evolution reaction. However, observing the course and slope of the curve for the unmodified ROY material, the recorded slope for Pd@ROY is significantly higher. The plotting of the slope of the Tafel in the selected range encounters a difficulty due to the presence of a marked increase in the recorded current from the electrical capacitance of the double layer, so extrapolation of the results was necessary for Pt@ROY and Pd-Pt@ROY. The results obtained for Pt@ROY and Pd-Pt@ROY are 191 mV dec^−1^ and 156 mV dec^−1^, respectively, which is a much better result, demonstrating the high electrochemical activity of the obtained platinum-based materials. The results obtained are a result of the very high surface development of the ROY material and the presence of nanometric particles of platinum and platinum-palladium alloy both inside the porous material and on its surface.

## 5. Conclusions

The presented works and obtained results are promising in the context of W4P concept development. It was shown that diluted aqueous solution can be an ideal composition for single and bimetallic catalyst synthesis. The application of activated carbon (ROY 0.8) works both as an efficient metal sorbent as well as a catalyst carrier. From kinetic curves, registered during the adsorption of Pd(II) and Pt(IV) ions on AC, the values of the rate constants were determined. The process runs in the order Pd-Pt@ROY > Pt@ROY > Pd@ROY. IR and TGA analysis indicates that the carbon surface is reached in carboxylic and phenolic groups.

The morphology of deposited Pd on AC was dependent of chloride ions, which promote pyramide-like structure formation. The mixture of Pd(II) and Pt(IV) ions led to the formation of a bimetallic structure, in which Pt nanoparticles “doped” Pd nanopyramids. The chemical composition and the obtained morphology strongly affect the catalytic activity of the material. The highest activity was recorded for materials with uniformly distributed Pd and nanopyramidal palladium structures enriched with platinum nanoparticles. The obtained results strongly indicate the significance of synthesis parameters to the final properties of synthesized materials in terms of their catalytic applications.

## Figures and Tables

**Table 1:** Kinetic rate constants determined for the adsorption process carried out for solutions containing Pd(II) and Pt(IV) as well as a mixture of these ions. Conditions: m_ROY_ = 0.5 g, C_0, Pd(II), Pt(IV)_ = 5 × 10^−4^ mol/dm^3^, V = 50 mL, T = 20 °C, 500 rpm, time = 25 min, interval = 5 min.

	Samples		
	Pd@ROY	Pt@ROY	Pd-Pt@ROY
Rate constant, min^−1^	0.195	0.238	0.278

**Table 2:** Structural Raman parameters.

Sample	I_D_/I_G_	I_D2_/I_G2_	La I_D_/I_G_	La I_D2_/I_G2_
	a.u.	a.u.	nm	nm
ROY	1.271	1.013	15.13	18.98
Pt@ROY	1.256	1.013	15.31	19.22
Pd@ROY	1.228	0.953	15.66	20.18
Pd-Pt@ROY	1.207	0.827	15.93	23.25

**Table 3:** XRD peaks from (1 1 1) and (2 0 0) planes of the experimental sample, ICDD cards, and the literature data. ACF—Activated Carbon Fibers.

Plane	Sample	2 Θ, °	
		Experimental	ICDD Cards/Ref.
	Pt		39.956 ^1^
(1 1 1)	Pt-Pd@ACF	40.035	[4]
	Pd-Pt@ROY	40.042	This work
	Pd		40.47 ^2^
	Pd@ACF	40.110	[4]
	Pd@ROY	40.333	This work
	Pt		46.472 ^1^
(2 0 0)	Pt-Pd@ACF	46.629	[4]
	Pd-Pt@ROY	46.621	This work
	Pd		46.88 ^2^
	Pd@ACF	46.684	[4]
	Pd@ROY	46.855	This work

^1^ ICCD card: 04-001-3301; ^2^ ICCD card: 01-087-06451.

## Data Availability

Data are contained within the article and Supplementary Materials.

## Supplementary Materials

The following supporting information can be downloaded at https://www.mdpi.com/article/10.3390/ma17164165/s1: Figure S1. Spectra for ROY sample containing only chloride ions (ROY_Cl) at different times of the adsorption process. Conditions: mROY = 0.5 g, C_0, Cl^−^_ ~3 mmol/dm^3^, V = 50 mL, 500 rpm, T = 20 °C. Table S1. Deconvolution parameters. Figure S2. Deconvoluted Raman spectra of ROY (a) and Pd-Pt@ROY (b) samples. Figure S3. IR analysis was performed for ROY, Pd@ROY, Pt@ROY, and Pd-Pt@ROY samples with characteristic peaks. Figure S4. TGA analysis of Pd@ROY sample with marked interesting area (grey) (a); Frist derivative of temperature difference for Pd@ROY (b), Pt@ROY (c), and Pd-Pt@ROY (d). Figure S5. Cross-section of the obtained materials at different magnifications. Samples: Pd@ROY (a, a’); Pt@ROY (b, b’); and Pd-Pt@ROY (c, c’). Figure S6. The SEM micrographs of Pt@ROY surface (a) and EDS analysis at selected areas (1, 2, and 3) (b, c, and d), respectively. Table S2. The EDS analysis of the Pt@ROY surface at selected areas 1, 2, and 3. Figure S7. The SEM micrographs of the Pt@ROY cross-section (a) and EDS analysis at selected area (1) (b). Table S3. The EDS analysis of the Pt@ROY surface (a) at selected area 1. Figure S8. SEM analysis of Pd@ROY at different magnifications (a–c). Figure S9. The SEM micrographs of Pd@ROY surface (a) and EDS analysis at selected areas (1 and 2) (b and c), respectively. Table S4. The EDS analysis of Pd@ROY surface at selected areas 1 and 2. Figure S10. The SEM micrographs of Pd@ROY cross-section (a) and EDS analysis at selected area (1) (b). Table S5. The EDS analysis of Pd@ROY cross-section at the selected area. Figure S11. The SEM micrographs of Pd-Pt@ROY surface (a) and EDS analysis at selected area (1) (b). Table S6. The EDS analysis of Pd-Pt@ROY surface (a) at selected area 1. Figure S12. The SEM micrographs of Pd-Pt@ROY surface (a) and EDS analysis at selected points (1 and 2) (b and c) and area (3) (d), respectively. Table S7. The EDS analysis of Pd-Pt@ROY surface (a) selected points (1 and 2) (b and c) and area (3) (d), respectively. Figure S13. The SEM micrographs of the Pd-Pt@ROY cross-section (a) and EDS analysis at selected areas (1 and 2) (b and c). Table S8. The EDS analysis of Pd-Pt@ROY cross-section (a) at selected areas (1 and 2) (b and c), respectively. Figure S14. SEM analysis of Pd-Pt@ROY sample with selected points (yellow arrow). Figure S15. The SEM micrographs of Pd-Pt@ROY nanopyramid and marked direction of analysis (arrow) (a) and EDS profile at the selected line (b). Figure S16. The SEM micrographs of Pd-Pt@ROY nanopyramid and marked direction of analysis (arrow) (a) and EDS profile at the selected line (b). Figure S17. TGA of Pd@ROY_Cl (a); ROY_Cl samples (b); first derivative of temperature difference for Pd@ROY_Cl (c) and ROY_Cl (d) samples. Notation: H_2_O_ads—_adsorbed water. Figure S18. XRD patterns for ROY, Pd@ROY, Pd-Pt@ROY, and Pt@ROY samples (a) * denote peaks location characteristic for Pd; Fragment of XRD pattern for Pd@ROY sample and peak deconvolution using Gaussian mode in Origin software, and peaks assignments: 40.042, 43.589, and 46.621° (b); Fragment of XRD pattern for the Pd-Pt@ROY sample and peak deconvolution using Gaussian mode in Origin software and peaks assignments: 40.333, 43.741, and 46.855° (c).

## References

[1] Briffa J., Sinagra E., Blundell R. (2020). Heavy metal pollution in the environment and their toxicological effects on humans. Heliyon.

[2] Myriam El A.-H., Fayçal H., Takemi O. (2021). Heavy Metals in the Environment and Health Impact. Environmental Health.

[3] Tchounwou P.B., Yedjou C.G., Patlolla A.K., Sutton D.J. (2012). Heavy metal toxicity and the environment. Molecular, Clinical and Environmental Toxicology.

[4] Pach A., Zaryczny A., Michałek T., Kamiński H., Kutyła D., Tokarski T., Chat-Wilk K., Hessel V., Luty-Błocho M. (2024). One-Step Synthesis of Pt–Pd@ACF Catalyst in the Microreactor System for the Hydrogen Evolution Reaction. Ind. Eng. Chem. Res..

[5] Xiang H., Min X., Tang C.-J., Sillanpää M., Zhao F. (2022). Recent advances in membrane filtration for heavy metal removal from wastewater: A mini review. J. Water Process Eng..

[6] Silva J.E., Paiva A.P., Soares D., Labrincha A., Castro F. (2005). Solvent extraction applied to the recovery of heavy metals from galvanic sludge. J. Hazard. Mater..

[7] Gulliani S., Volpe M., Messineo A., Volpe R. (2023). Recovery of metals and valuable chemicals from waste electric and electronic materials: A critical review of existing technologies. RSC Sustain..

[8] Qasem N.A.A., Mohammed R.H., Lawal D.U. (2021). Removal of heavy metal ions from wastewater: A comprehensive and critical review. NPJ Clean Water.

[9] Fei Y., Hu Y.H. (2022). Design, synthesis, and performance of adsorbents for heavy metal removal from wastewater: A review. J. Mater. Chem. A.

[10] Mariana M., H.P.S A.K., Mistar E.M., Yahya E.B., Alfatah T., Danish M., Amayreh M. (2021). Recent advances in activated carbon modification techniques for enhanced heavy metal adsorption. J. Water Process Eng..

[11] George R., Bahadur N., Singh N., Singh R., Verma A., Shukla A.K. (2016). Environmentally Benign TiO_2_ Nanomaterials for Removal of Heavy Metal Ions with Interfering Ions Present in Tap Water. Mater. Today Proc..

[12] Yang J., Liu S., Xu X., Pan B., Long Q., Cheng L., Deng J., Yao Q., Lu Z., Wang Z. (2023). Mechanism of Pd(II) adsorption by nanoscale titanium dioxide loaded bamboo shoot shell biomass. Environ. Sci. Pollut. Res. Int..

[13] Chen B., Li L., Liu L., Cao J. (2023). Effective adsorption of heavy metal ions in water by sulfhydryl modified nano titanium dioxide. Front. Chem..

[14] Zhao X., Jia Q., Song N., Zhou W., Li Y. (2010). Adsorption of Pb(II) from an Aqueous Solution by Titanium Dioxide/Carbon Nanotube Nanocomposites: Kinetics, Thermodynamics, and Isotherms. J. Chem. Eng. Data.

[15] Akpomie K.G., Conradie J., Adegoke K.A., Oyedotun K.O., Ighalo J.O., Amaku J.F., Olisah C., Adeola A.O., Iwuozor K.O. (2022). Adsorption mechanism and modeling of radionuclides and heavy metals onto ZnO nanoparticles: A review. Appl. Water Sci..

[16] Le A.T., Pung S.-Y., Sreekantan S., Matsuda A., Huynh D.P. (2019). Mechanisms of removal of heavy metal ions by ZnO particles. Heliyon.

[17] Saad A.H.A., Azzam A.M., El-Wakeel S.T., Mostafa B.B., Abd El-latif M.B. (2018). Removal of toxic metal ions from wastewater using ZnO@Chitosan core-shell nanocomposite. Environ. Nanotechnol. Monit. Manag..

[18] Alanazi A.G., Habila M.A., Alothman Z.A., Badjah-Hadj-Ahmed A.-Y. (2024). Synthesis and Characterization of Zinc Oxide Nanoparticle Anchored Carbon as Hybrid Adsorbent Materials for Effective Heavy Metals Uptake from Wastewater. Crystals.

[19] Haripriyan U., Gopinath K.P., Arun J. (2022). Chitosan based nano adsorbents and its types for heavy metal removal: A mini review. Mater. Lett..

[20] Boukhlifi F., Mario Alfonso M.-T., Hugo S.-N., Agnieszka S. (2021). Sustainable Treatment of Heavy Metals by Adsorption on Raw Chitin/Chitosan. Trace Metals in the Environment.

[21] Trikkaliotis D.G., Ainali N.M., Tolkou A.K., Mitropoulos A.C., Lambropoulou D.A., Bikiaris D.N., Kyzas G.Z. (2022). Removal of Heavy Metal Ions from Wastewaters by Using Chitosan/Poly(Vinyl Alcohol) Adsorbents: A Review. Macromol.

[22] Wang K., Zhang F., Xu K., Che Y., Qi M., Song C. (2023). Modified magnetic chitosan materials for heavy metal adsorption: A review. RSC Adv..

[23] Duan C., Ma T., Wang J., Zhou Y. (2020). Removal of heavy metals from aqueous solution using carbon-based adsorbents: A review. J. Water Process Eng..

[24] Wang B., Lan J., Bo C., Gong B., Ou J. (2023). Adsorption of heavy metal onto biomass-derived activated carbon: Review. RSC Adv..

[25] Sheraz N., Shah A., Haleem A., Iftikhar F.J. (2024). Comprehensive assessment of carbon-, biomaterial- and inorganic-based adsorbents for the removal of the most hazardous heavy metal ions from wastewater. RSC Adv..

[26] Jüntgen H. (1986). Activated carbon as catalyst support: A review of new research results. Fuel.

[27] Iwanow M., Gärtner T., Sieber V., König B. (2020). Activated carbon as catalyst support: Precursors, preparation, modification and characterization. Beilstein J. Org. Chem..

[28] Wojnicki M., Socha R.P., Pędzich Z., Mech K., Tokarski T., Fitzner K. (2018). Palladium(II) Chloride Complex Ion Recovery from Aqueous Solutions Using Adsorption on Activated Carbon. J. Chem. Eng. Data.

[29] Wojnicki M., Fitzner K. (2018). Kinetic modeling of the adsorption process of Pd(II) complex ions onto activated carbon. React. Kinet. Mech. Catal..

[30] Wojnicki M., PacŁAwski K., Socha R.P., Fitzner K. (2013). Adsorption and reduction of platinum(IV) chloride complex ions on activated carbon. Trans. Nonferrous Met. Soc. China.

[31] Wojnicki M., Rudnik E., Socha R.P., Fitzner K. (2017). Platinum(IV) Chloride Complex Ions Adsorption on Activated Carbon Organosorb 10CO. Aust. J. Chem..

[32] Wojnicki M., Socha R.P., Luty-Błocho M., Fitzner K. (2017). Kinetic studies of the removal of Pt(IV) chloride complex ions from acidic aqueous solutions using activated carbon. React. Kinet. Mech. Catal..

[33] Michałek T., Wojtaszek K., Małecki S., Kornaus K., Wandor S., Druciarek J., Fitzner K., Wojnicki M. (2023). Recovery of Pd(II) Ions from Aqueous Solutions Using Activated Carbon Obtained in a Single-Stage Synthesis from Cherry Seeds. C.

[34] Gurrath M., Kuretzky T., Boehm H.P., Okhlopkova L.B., Lisitsyn A.S., Likholobov V.A. (2000). Palladium catalysts on activated carbon supports: Influence of reduction temperature, origin of the support and pretreatments of the carbon surface. Carbon.

[35] Luty-Błocho M., Wojnicki M., Włoch G., Fitzner K. (2018). Green method for efficient PdNPs deposition on carbon carrier in the microreactor system. J. Nanopart. Res..

[36] Luty-Błocho M., Wojnicki M., Pacławski K., Fitzner K. (2013). The synthesis of platinum nanoparticles and their deposition on the active carbon fibers in one microreactor cycle. Chem. Eng. J..

[37] Pikna Ľ., Milkovič O., Saksl K., Heželová M., Smrčová M., Puliš P., Michalik Š., Gamcová J. (2014). The structure of nano-palladium deposited on carbon-based supports. J. Solid State Chem..

[38] Luty-Błocho M. (2023). The influence of steric stabilization on process of Au, Pt nanoparticles formation. Arch. Metall. Mater..

[39] Sharififard H., Soleimani M., Zokaee Ashtiani F. (2016). Application of nanoscale iron oxide-hydroxide-impregnated activated carbon (Fe-AC) as an adsorbent for vanadium recovery from aqueous solutions. Desalination Water Treat..

[40] Ho Kim Y., Nakano Y. (2005). Adsorption mechanism of palladium by redox within condensed-tannin gel. Water Res..

[41] Wojnicki M., Podborska A. (2017). The Mechanism of Redox Reaction between Palladium(II) Complex Ions and Potassium Formate in Acidic Aqueous Solution. Arch. Metall. Mater..

[42] Sun Z., Chai L., Shu Y., Li Q., Liu M., Qiu D. (2017). Chemical bond between chloride ions and surface carboxyl groups on activated carbon. Colloids Surf. A Physicochem. Eng. Asp..

[43] Simonov P.A., Moroz E.M., Chuvilin A.L., Kolomiichuk V.N., Boronin A.I., Likholobov V.A., Poncelet G., Martens J., Delmon B., Jacobs P.A., Grange P. (1995). Influence of an interaction of PdCl2 with carbon support on state and catalytic properties of Pd/C catalysts. Studies in Surface Science and Catalysis.

[44] Luty-Błocho M., Wojnicki M., Csapo E., Fitzner K. (2021). On the Rate of Interaction of Sodium Borohydride with Platinum (IV) Chloride Complexes in Alkaline Media. Materials.

[45] Simonov P.A., Romanenko A.V., Prosvirin I.P., Moroz E.M., Boronin A.I., Chuvilin A.L., Likholobov V.A. (1997). On the nature of the interaction of H2PdCl4 with the surface of graphite-like carbon materials. Carbon.

[46] Higashi N., Ozaki Y. (2004). Potential of far-ultraviolet absorption spectroscopy as a highly sensitive quantitative and qualitative analysis method for aqueous solutions, part I: Determination of hydrogen chloride in aqueous solutions. Appl. Spectrosc..

[47] Boily J.-F., Seward T.M. (2005). Palladium(II) chloride complexation: Spectrophotometric investigation in aqueous solutions from 5 to 125°C and theoretical insight into Pd-Cl and Pd-OH2 interactions. Geochim. Cosmochim. Acta.

[48] Saito S., Numadate N., Teraoka H., Enami S., Kobayashi H., Hama T. (2023). Impurity contribution to ultraviolet absorption of saturated fatty acids. Sci. Adv..

[49] Coyle J.D. (1978). Photochemistry of carboxylic acid derivatives. Chem. Rev..

[50] Kaeswurm J.A.H., Scharinger A., Teipel J., Buchweitz M. (2021). Absorption Coefficients of Phenolic Structures in Different Solvents Routinely Used for Experiments. Molecules.

[51] Shimodaira N., Masui A. (2002). Raman spectroscopic investigations of activated carbon materials. J. Appl. Phys..

[52] Xing Z., Qi Y., Tian Z., Xu J., Yuan Y., Bommier C., Lu J., Tong W., Jiang D.-E., Ji X. (2017). Identify the Removable Substructure in Carbon Activation. Chem. Mater..

[53] Bokobza L., Bruneel J.-L., Couzi M. (2015). Raman Spectra of Carbon-Based Materials (from Graphite to Carbon Black) and of Some Silicone Composites. C.

[54] Zambrzycki M., Jeleń P., Fraczek-Szczypta A. (2022). Structure and electrical transport properties of electrospun carbon nanofibers/carbon nanotubes 3D hierarchical nanocomposites: Effect of the CCVD synthesis conditions. J. Mater. Sci..

[55] Cançado L.G., Takai K., Enoki T., Endo M., Kim Y.A., Mizusaki H., Jorio A., Coelho L.N., Magalhães-Paniago R., Pimenta M.A. (2006). General equation for the determination of the crystallite size La of nanographite by Raman spectroscopy. Appl. Phys. Lett..

[56] Cansado I.P., Mourão P.A., Castanheiro J.E. (2023). Performance of Regenerated Activated Carbons on Pesticides Removal from the Aqueous Phase. Processes.

[57] Azhagapillai P., Al Shoaibi A., Chandrasekar S. (2021). Surface functionalization methodologies on activated carbons and their benzene adsorption. Carbon Lett..

[58] Rashidi N.A., Bokhari A., Yusup S. (2021). Evaluation of kinetics and mechanism properties of CO2 adsorption onto the palm kernel shell activated carbon. Environ. Sci. Pollut. Res..

[59] Pudney P.D.A., Mutch K.J., Zhu S. (2009). Characterising the phase behaviour of stearic acid and its triethanolamine soap and acid–soap by infrared spectroscopy. Phys. Chem. Chem. Phys..

[60] Squeo G., Grassi S., Paradiso V.M., Alamprese C., Caponio F. (2019). FT-IR extra virgin olive oil classification based on ethyl ester content. Food Control.

[61] De la Puente G., Pis J.J., Menéndez J.A., Grange P. (1997). Thermal stability of oxygenated functions in activated carbons. J. Anal. Appl. Pyrolysis.

[62] Skibińska K., Żabiński P. (2024). Nanocones: A Compressive Review of Their Electrochemical Synthesis and Applications. Materials.

[63] Wang Y., Zhang G., He Z., Chen J., Gao W., Cao P. (2023). Superhydrophobic Ni nanocone surface prepared by electrodeposition and its overall performance. Surf. Coat. Technol..

[64] Penki T.R., Shanmughasundaram D., Kishore B., Munichandraiah N. (2014). High rate capability of coconut kernel derived carbon as an anode material for lithium-ion batteries. Adv. Mat. Lett..

