# Supplementary material for: Waste for Product—Synthesis and Electrocatalytic Properties of Palladium Nanopyramid Layer Enriched with PtNPs
Source: Materials (Basel). 2024 Aug 22;17(16):4165. doi: 10.3390/ma17164165 (PMC11356389; doi:10.3390/ma17164165)
Supplement: Supplementary file 1 [file materials-17-04165-s001.zip › materials-3139517-supplementary.pdf]

Supporting Materials

Waste for Product—Synthesis and Electrocatalytic Properties of Palladium Nanopyramid Layer Enriched with PtNPs

Magdalena Luty-Błocho [1,*], Adrianna Pach [1], Dawid Kutyła [1], Anna Kula [1], Stanisław Małecki [1], Piotr Jeleń [2] and Volker Hessel [3]

[1] AGH University of Krakow, Faculty of Non-Ferrous Metals, Al. A. Mickiewicza 30, 30-059 Krakow, Poland
[2] AGH University of Krakow, Faculty of Materials Science and Ceramics, Al. A. Mickiewicza 30, 30-059 Krakow, Poland
[3] School of Chemical Engineering, University of Adelaide, Adelaide 5005, Australia
* Correspondence: mlb@agh.edu.pl

1. UV-Vis spectra for solution containing chloride ions.

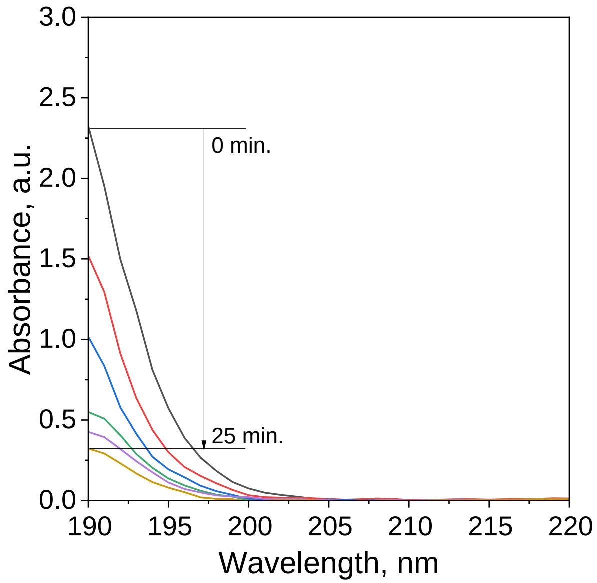

Figure S1. Spectra for ROY sample containing only chloride ions (ROY_Cl) at different time of adsorption process. Conditions: m_{ROY} = 0.5 g, $C_{0,\,Cl^-}$~ 3 mmol/dm^3, V = 50 mL, 500 rpm, T = 20°C.

2. Results from Raman analysis

Table S1. Deconvolution parameters.

ROY		Pt@ROY		Pd@ROY		Pd-Pt@ROY	
**Position [cm^{-1}]**	Intensity [a.u.]	Position [cm^{-1}]	Intensity [a.u.]	Position [cm^{-1}]	Intensity [a.u.]	Position [cm^{-1}]	Intensity [a.u.]
**1171**	0.23	1169	0.22	1169	0.24	1169	0.23
**1300**	0.44	1298	0.49	1302	0.40	1297	0.45
**1343**	1.58	1346	1.57	1345	1.55	1349	1.54
**1560**	0.43	1558	0.48	1559	0.42	1557	0.54
**1604**	1.24	1605	1.25	1606	1.26	1606	1.27

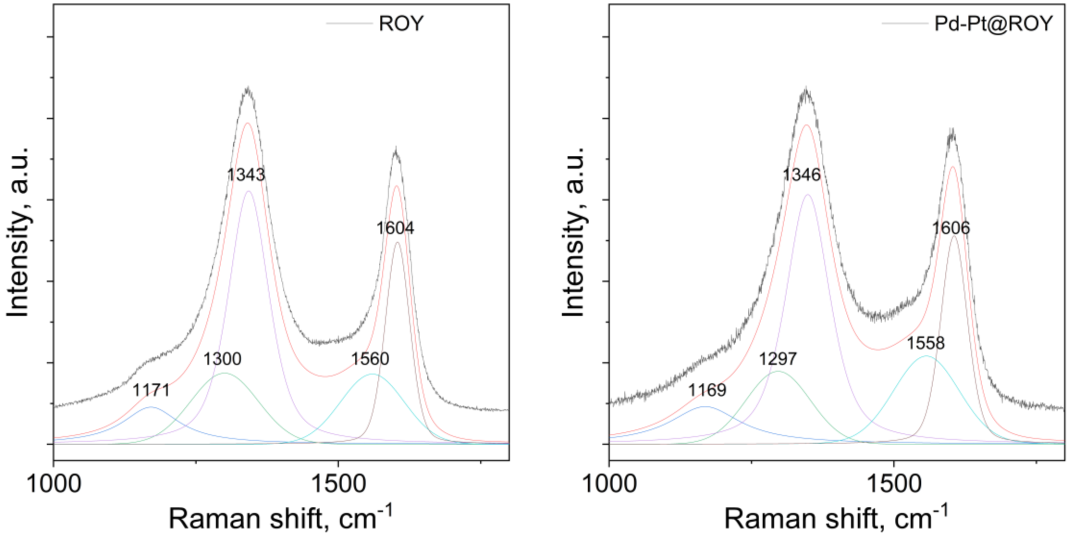

(a) **(b)**

Figure S2. Deconvoluted Raman spectra of ROY (a) and Pd-Pt@ROY (b) samples.

3. Detailed results from IR analysis.

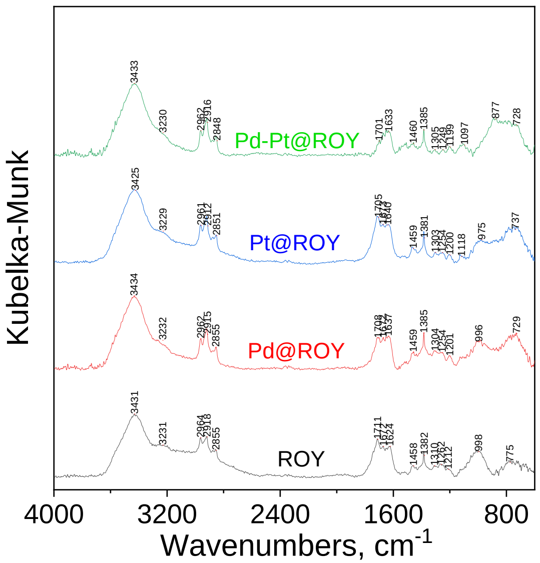

Figure S3. IR analysis performed for ROY, Pd@ROY, Pt@ROY and Pd-Pt@ROY samples with characteristic bands. .

4. TGA analysis

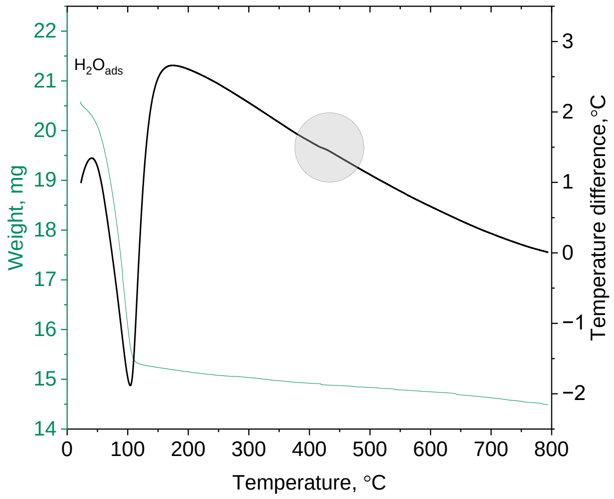

(a)

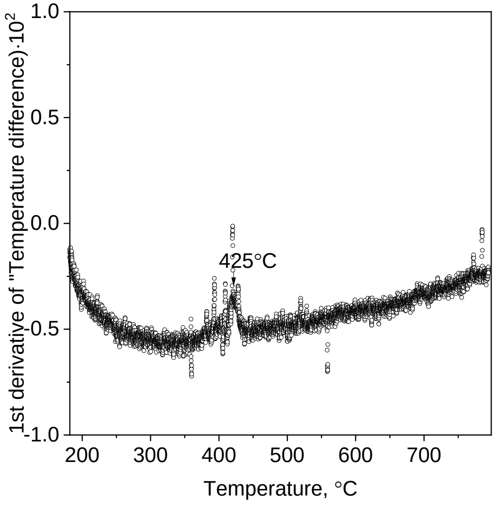

(b)

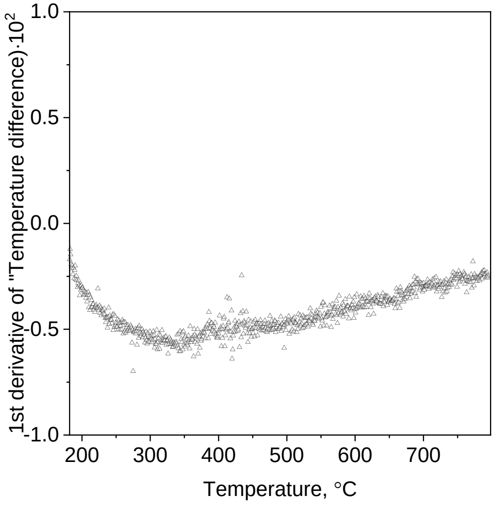

(c)

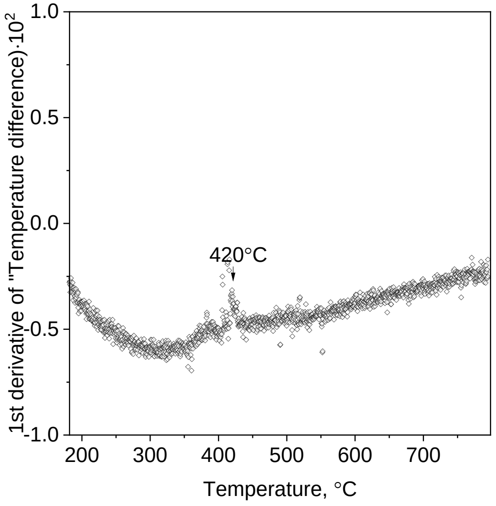

(d)

Figure S4. TGA analysis of Pd@ROY sample with marked interesting area (grey) (a); 1st derivative of temperature difference for Pd@ROY (b), Pt@ROY (c) and Pd-Pt@ROY (d).

5. Results from SEM/EDS analysis.

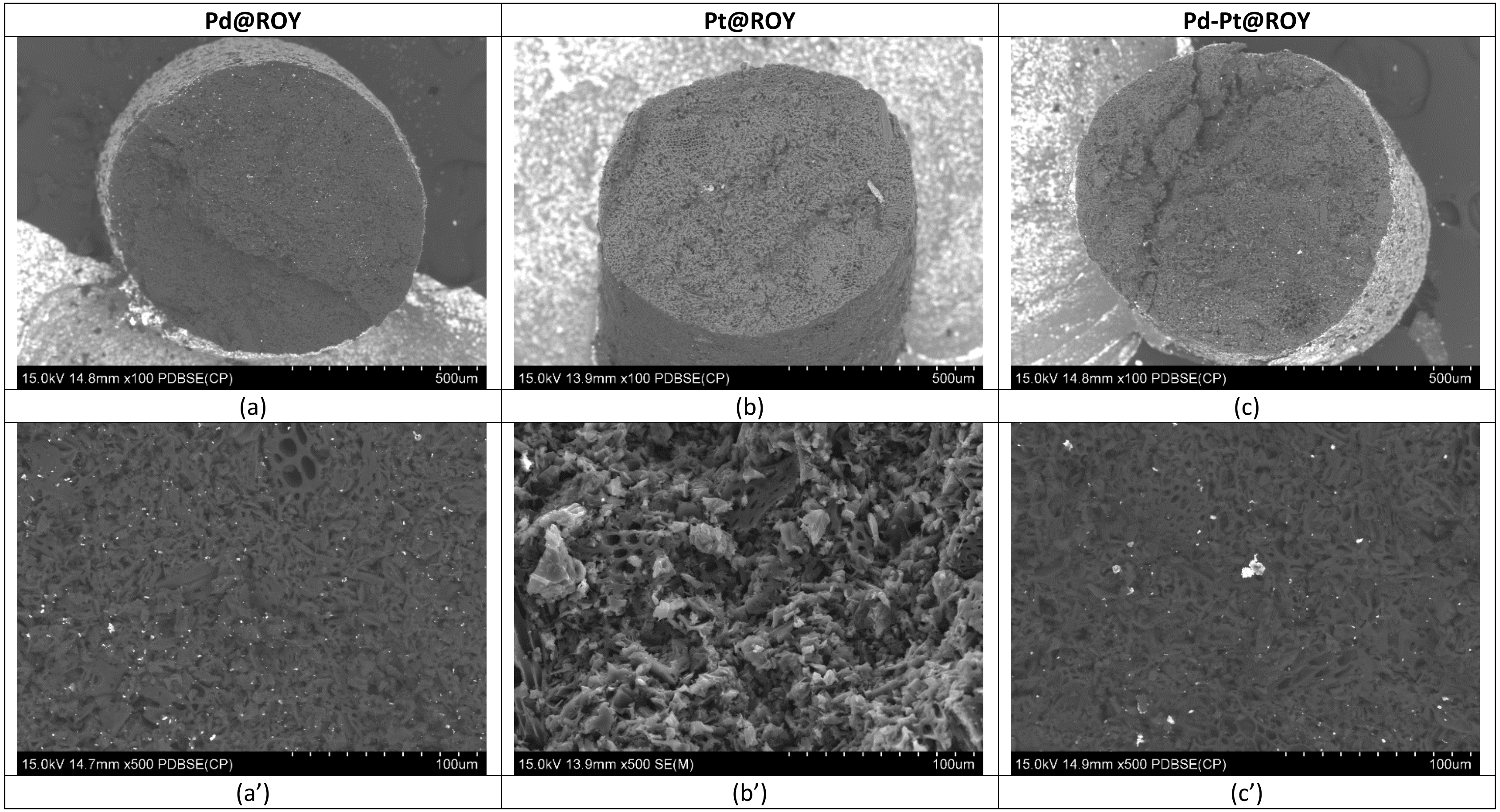

Figure S5. Cross section of the obtained materials at different magnifications. Samples: Pd@ROY (a, a’); Pt@ROY (b, b’); Pd-Pt@ROY (c, c’).

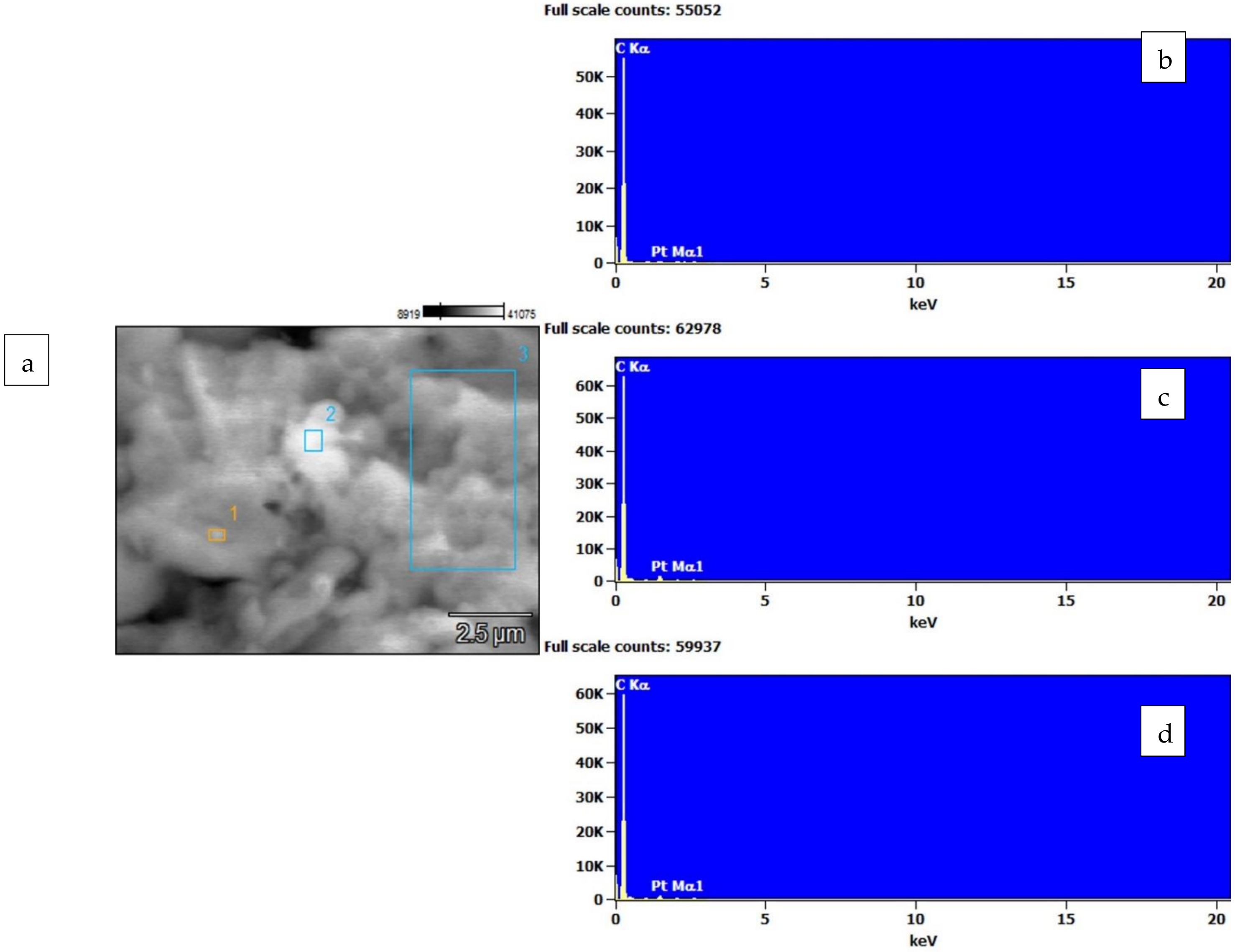

a
8919
41075
1
2
3
2.5 µm
Full scale counts: 55052
b
C Kα
Pt Mα1
keV
Full scale counts: 62978
c
C Kα
Pt Mα1
keV
Full scale counts: 59937
d
C Kα
Pt Mα1
keV

Figure S6. The SEM micrographs of Pt@ROY surface (a) and EDS analysis at selected area (1, 2, 3) (b, c, d), respectively.

Table S2. The EDS analysis of Pt@ROY surface at selected areas 1, 2,3.

Area	Element concentration [weight %]	
	**C-K**	**Pt-M**
surface 1	99.43	0.57
surface 2	99.61	0.39
surface 3	99.48	0.52
**Area**	**Element concentration [atom%]**	
	**C-K**	**Pt-M**
surface 1	99.96	0.04
surface 2	99.98	0.02
surface 3	99.97	0.03

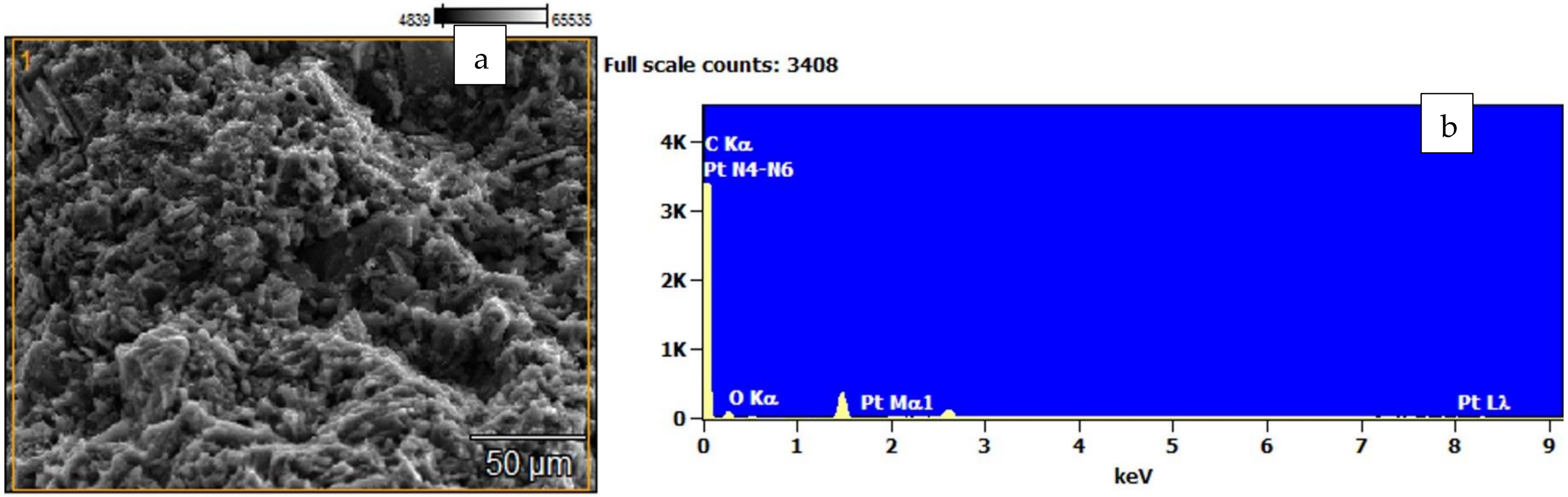

Figure S7. The SEM micrographs of Pt@ROY cross section (a) and EDS analysis at selected area (1) (b).

Table S3. The EDS analysis of Pt@ROY surface (a) at selected area 1.

Area	Element concentration [weight %]

	C-K	O-K	Pt-M
surface	55.65	33.38	10.96
**Area**	**Element concentration [atom %]**		
	**C-K**	**O-K**	**Pt-M**
surface	68.38	30.79	0.83

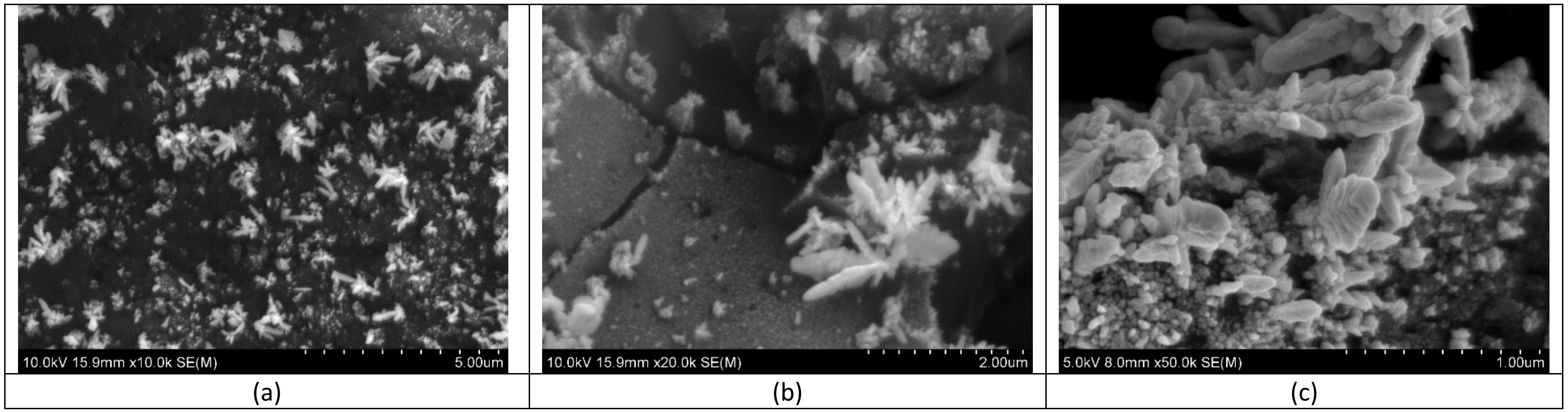

(a) (b) (c)

Figure S8. SEM analysis of Pd@ROY at different magnifications (a – c).

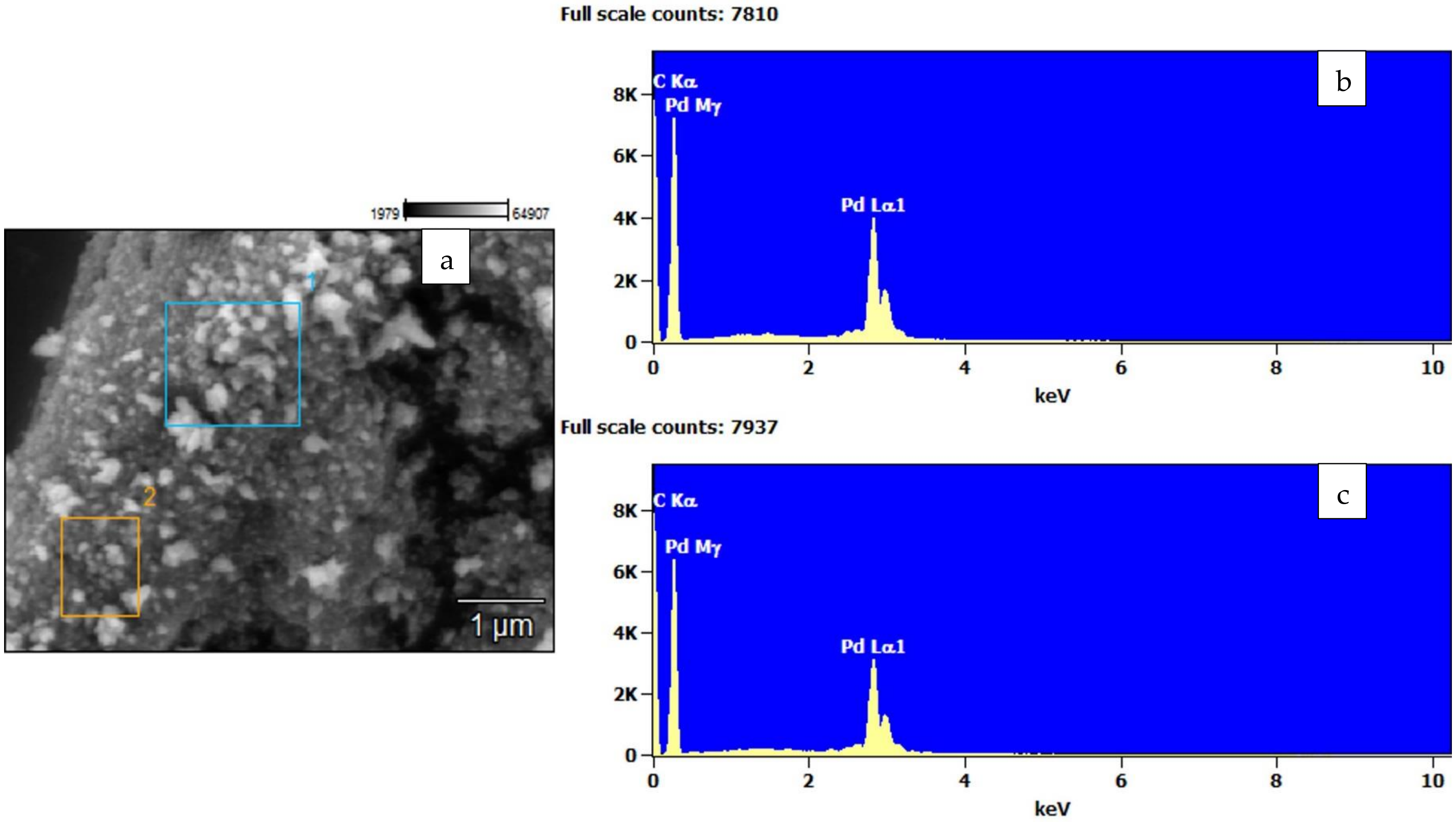

Figure S9. The SEM micrographs of Pd@ROY surface (a) and EDS analysis at selected area (1, 2) (b, c), respectively.

Table S4. The EDS analysis of Pd@ROY surface at selected areas 1, 2.

**Area**	**Element concentration [weight %]**		
	**C-K**	**O-K**	**Pd-L**
surface 1	26.40	0.20	73.40
surface 2	28.53	0.22	71.25
**Area**	**Element concentration [atom %]**		

	C-K	O-K	Pd-L
surface 1	75.97	0.43	23.78
surface 2	77.66	0.44	21.89

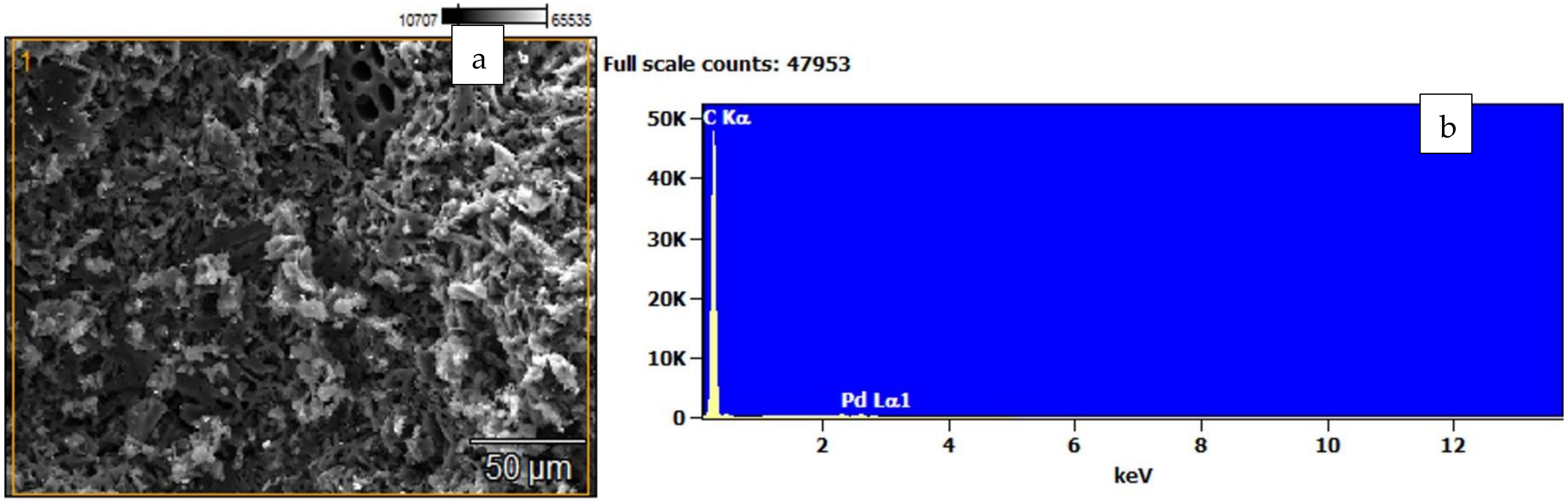

Figure S10. The SEM micrographs of Pd@ROY cross section (a) and EDS analysis at selected area (1) (b).

Table S5. The EDS analysis of Pd@ROY cross section at selected area.

Area	Element concentration [weight %]	
	**C-K**	**Pd-L**
cross section	98.91	1.09
**Area**	**Element concentration [atom %]**	
	**C-K**	**Pd-L**
cross section	99.88	0.12

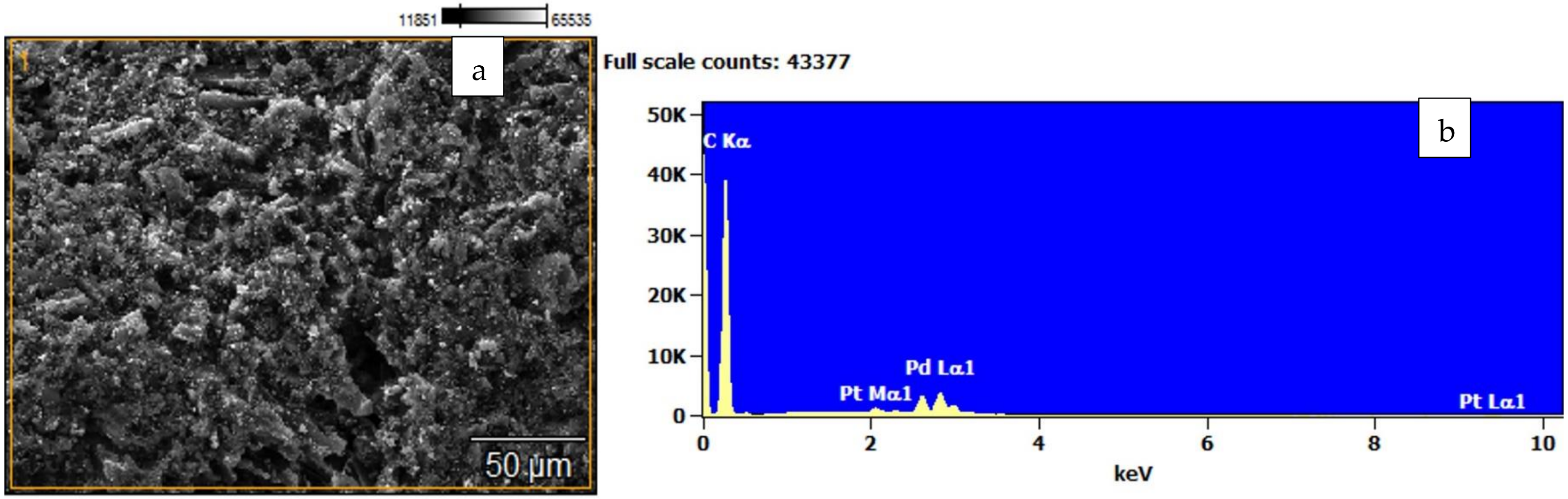

Figure S11. The SEM micrographs of Pd-Pt@ROY surface (a) and EDS analysis at selected area (1) (b).

Table S6. The EDS analysis of Pd-Pt@ROY surface (a) at selected area 1.

**Area**	**Element concentration [weight %]**		
	**C-K**	**Pd-L**	**Pt-L**
surface	89.14	7.28	3.58
**Area**	**Element concentration [weight %]**		
	**C-K**	**Pd-L**	**Pt-L**
surface	98.84	0.91	0.25

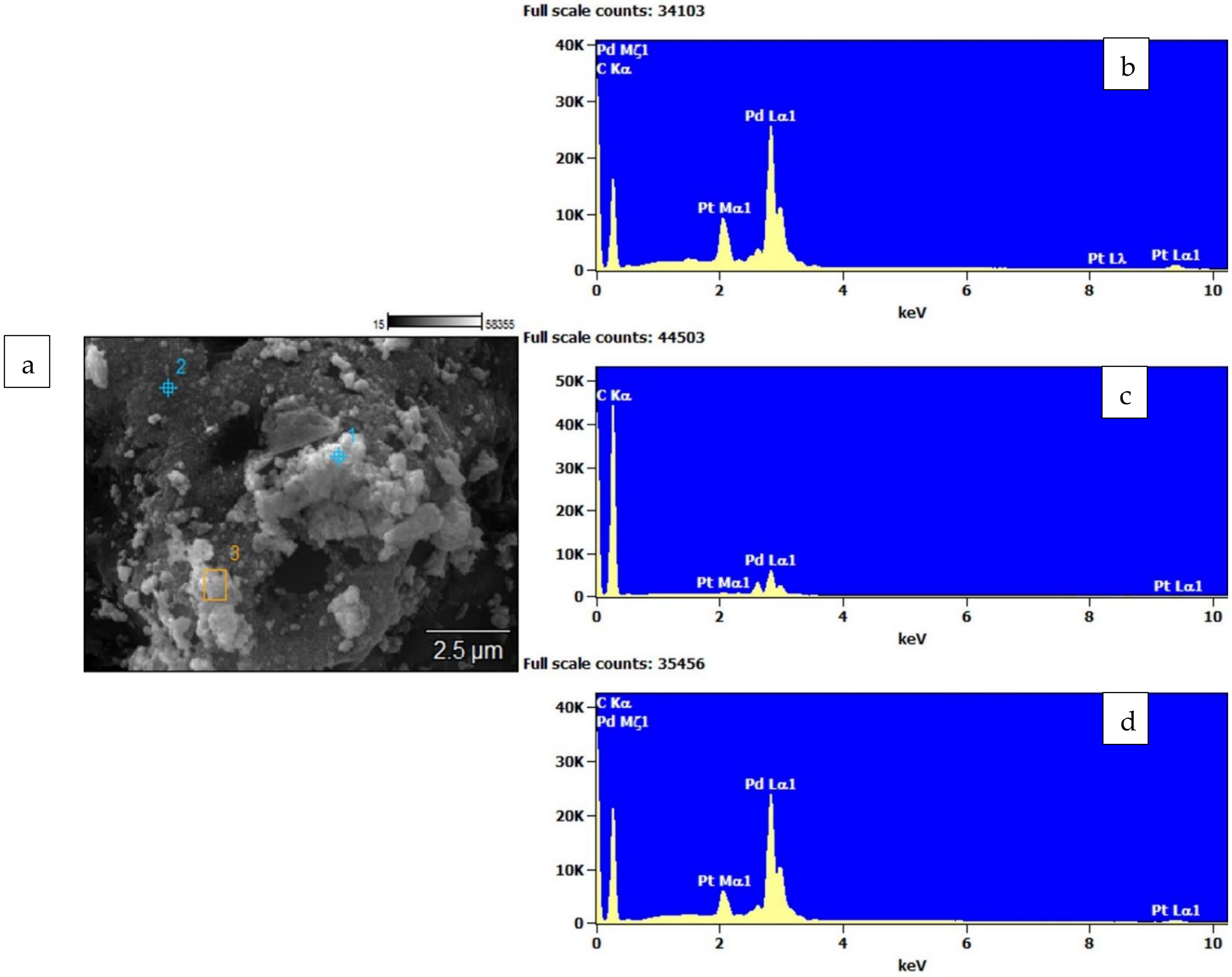

Figure S12. The SEM micrographs of Pd-Pt@ROY surface (a) and EDS analysis at selected point (1, 2) (b, c) and area (3) (d), respectively.

Table S7. The EDS analysis of Pd-Pt@ROY surface (a) selected point (1, 2) (b, c) and area (3) (d), respectively.

Area	Element concentration [weight %]		
	**C-K**	**Pd-L**	**Pt-L**
Point 1	34.30	47.93	17.77
Point 2	86.63	11.99	1.38
Surface 1	42.14	45.30	12.56
**Area**	**Element concentration [atom %]**		
	**C-K**	**Pd-L**	**Pt-L**
Point 1	84.06	13.26	2.68
Point 2	98.37	1.54	0.10
Surface 1	87.74	10.65	1.61

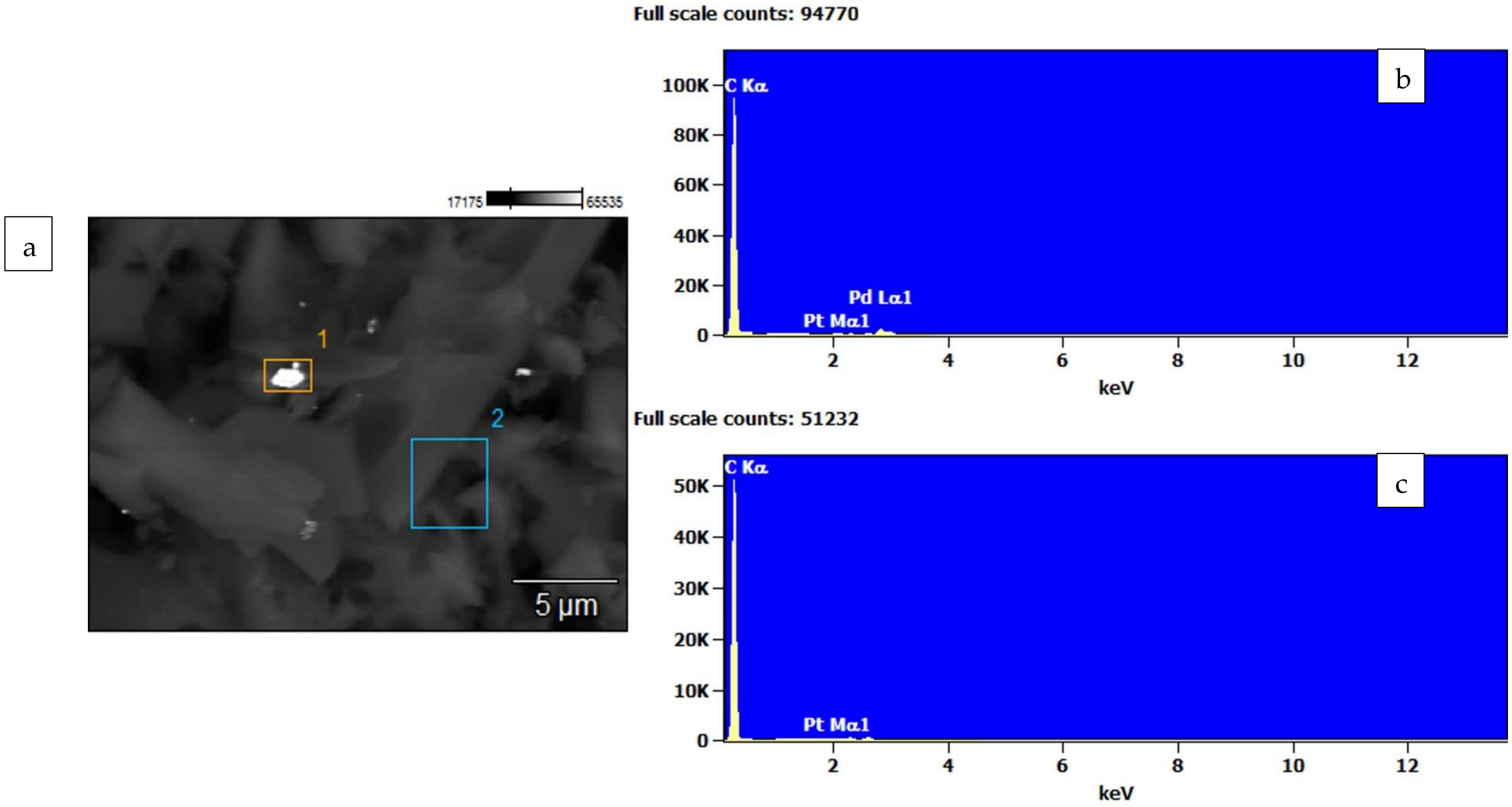

Figure S13. The SEM micrographs of Pd-Pt@ROY cross section (a) and EDS analysis at selected area (1, 2) (b, c).

Table S8. The EDS analysis of Pd-Pt@ROY cross section (a) at selected area (1, 2) (b, c), respectively.

**Area**	**Element concentration [weight %]**		
	**C-K**	**Pd-L**	**Pt-M**
Cross section 1	92.33	6.81	0.87
Cross section 2	99.33	-	0.67
	**C-K**	**Pd-L**	**Pt-M**

Cross section 1	99.12	0.82	0.06
Cross section 2	99.96	-	0.04

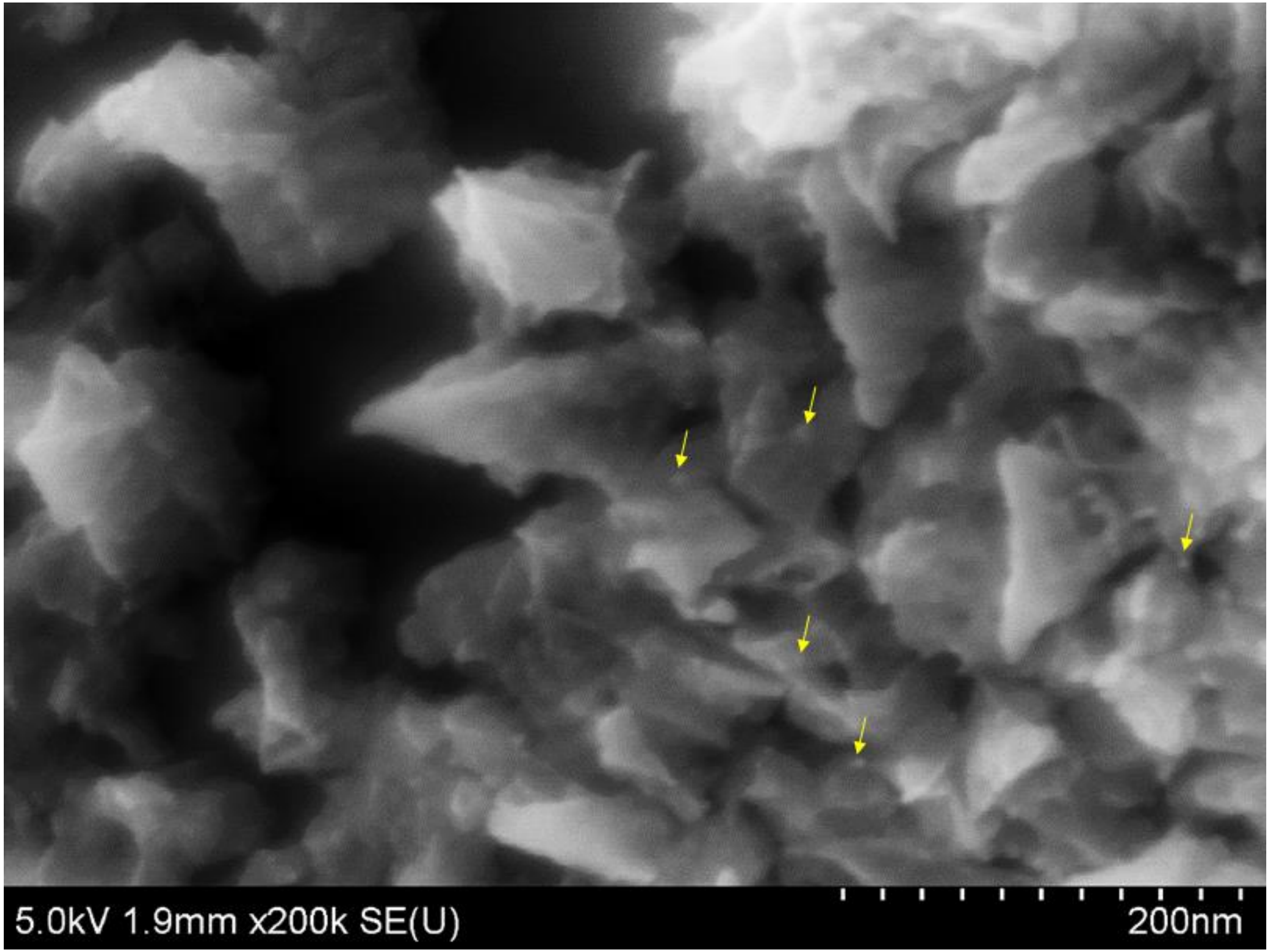

Figure S14. SEM analysis of Pd-Pt@ROY sample with selected points (PtNPs) (yellow arrow).

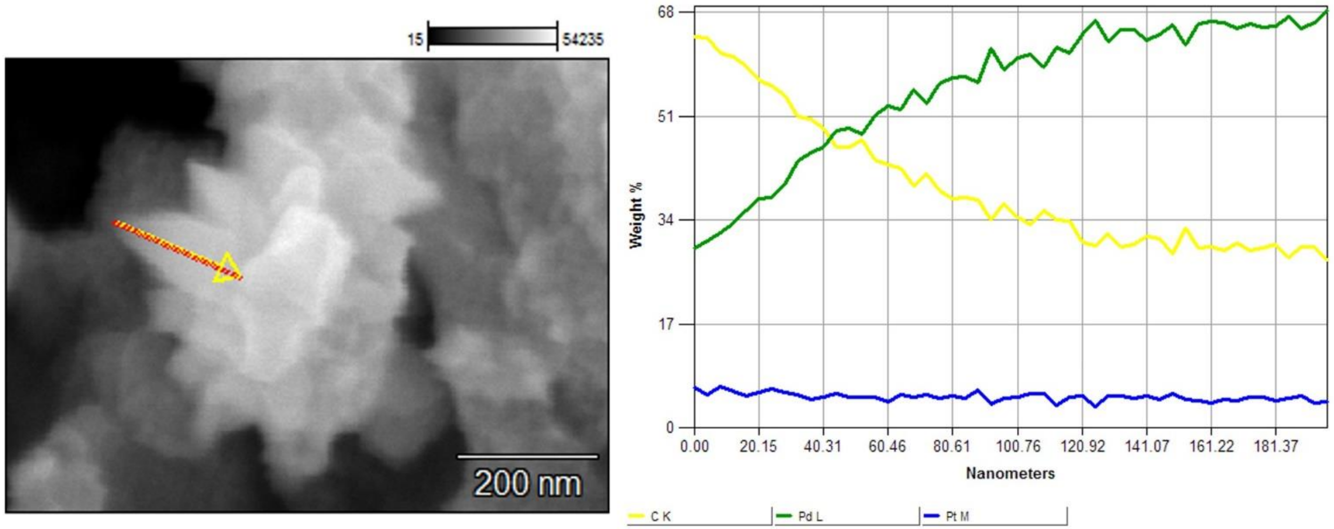

(a) (b)

Figure S15. The SEM micrographs of Pd-Pt@ROY nanopyramid and marked direction of analysis (arrow) (a) and EDS profile at selected line (b).

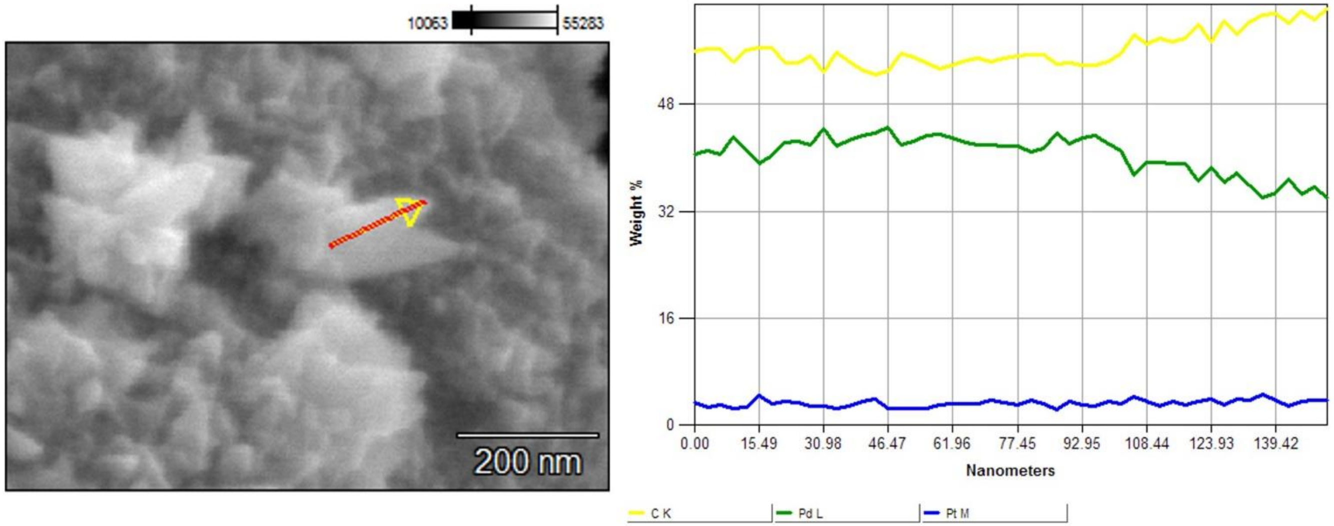

(a) (b)

Figure S16. The SEM micrographs of Pd-Pt@ROY nanopyramid and marked direction of analysis (arrow) (a) and EDS profile at selected line (b).

6. TGA analysis of Pd@ROY_Cl and ROY_Cl samples

Obtained results from TGA analysis for selected samples, i.e. Pd@ROY_Cl and ROY_Cl were shown in Fig. S17.

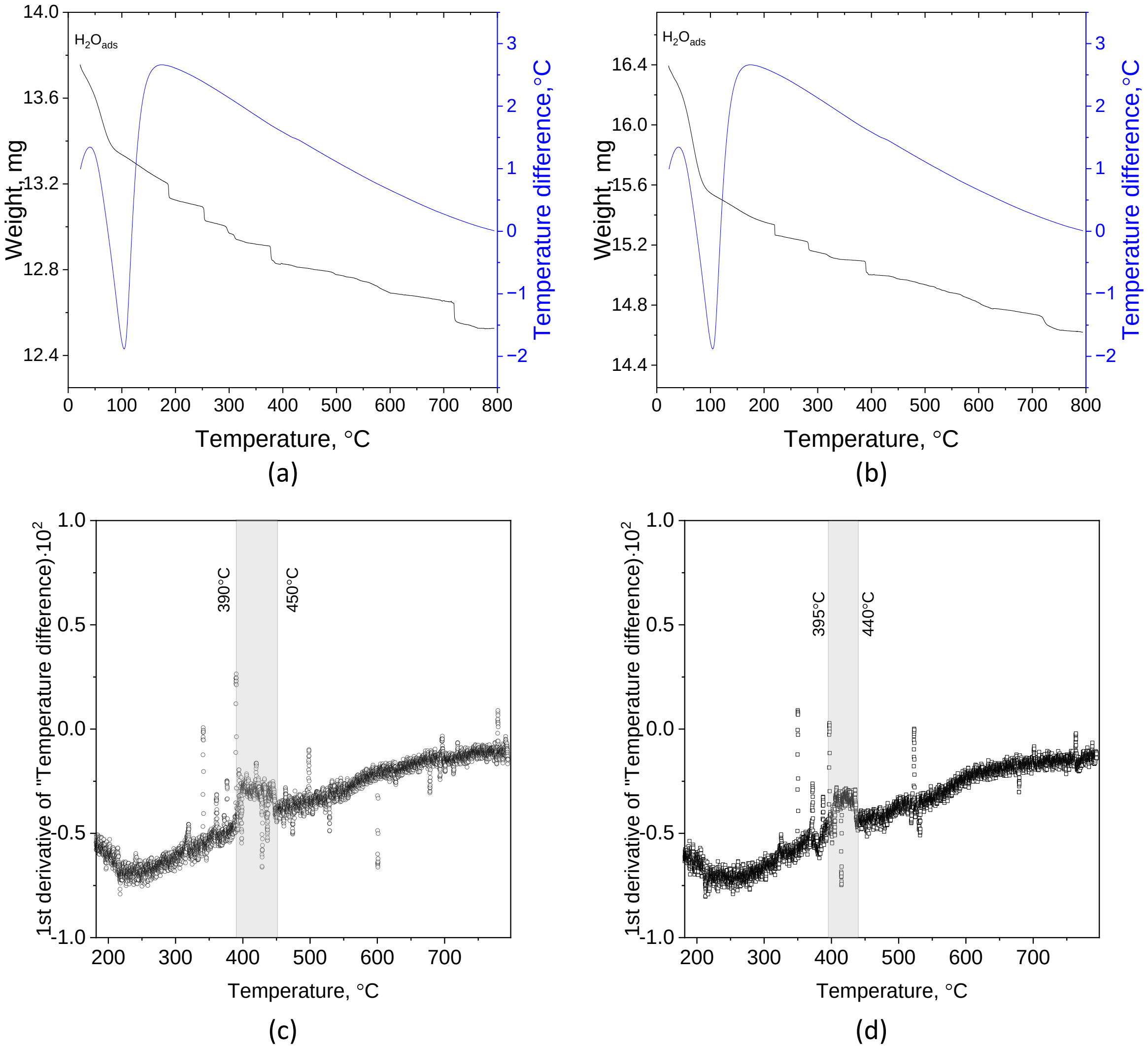

Figure S17. TGA of Pd@ROY_Cl (a); ROY_Cl samples (b); 1st derivative of temperature difference for Pd@ROY_Cl (c) and ROY_Cl (d) samples. Notation: H_20_{ads} – adsorbed water.

7. XRD analysis

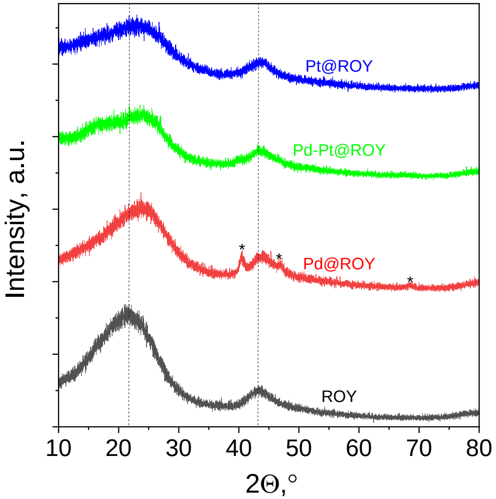

(a)

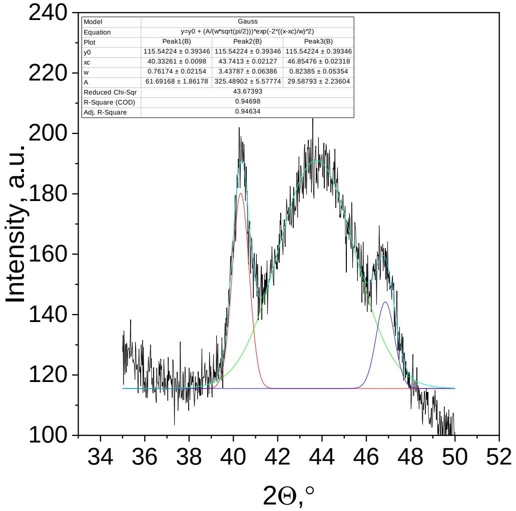

(b)

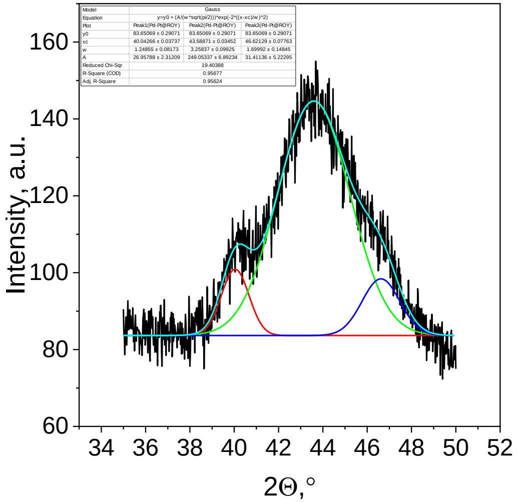

(c)

Figure S18. XRD patterns for ROY, Pd@ROY, Pd-Pt@ROY and Pt@ROY samples (a) * denote peaks location characteristic for Pd; Fragment of XRD pattern for Pd@ROY sample and peak deconvolution using Gaussian mode in Origin software, and peaks assignments: 40.042, 43.589 and 46.621° (b); Fragment of XRD pattern for Pd-Pt@ROY sample and peak deconvolution using Gaussian mode in Origin software and peaks assignments: 40.333, 43.741 and 46.855° (c).
